# Functional evaluation of *Bacillus licheniformis* PF9 for its potential in controlling enterotoxigenic *Escherichia coli* in weaned piglets

**DOI:** 10.1093/tas/txae050

**Published:** 2024-04-03

**Authors:** Haoxiang Xu, Joshua Gong, Peng Lu, Paula Azevedo, Linyan Li, Hai Yu, Chengbo Yang

**Affiliations:** Department of Animal Science, University of Manitoba, Winnipeg, Manitoba, CanadaR3T 2N2; Guelph Research and Development Centre, Agriculture Agri-Food Canada, Guelph, Ontario, CanadaN1G 5C9; Department of Animal Science, University of Manitoba, Winnipeg, Manitoba, CanadaR3T 2N2; Department of Animal Science, University of Manitoba, Winnipeg, Manitoba, CanadaR3T 2N2; Department of Animal Science, University of Manitoba, Winnipeg, Manitoba, CanadaR3T 2N2; Guelph Research and Development Centre, Agriculture Agri-Food Canada, Guelph, Ontario, CanadaN1G 5C9; State Key Laboratory of Food Science and Technology, Nanchang University, Nanchang 330047, China; Guelph Research and Development Centre, Agriculture Agri-Food Canada, Guelph, Ontario, CanadaN1G 5C9; Department of Animal Science, University of Manitoba, Winnipeg, Manitoba, CanadaR3T 2N2

**Keywords:** *Bacillus licheniformis* PF9, diarrhea, enterotoxigenic *E. coli*, gut health, weaned piglets

## Abstract

During the bacterial selection, isolate PF9 demonstrated tolerance to low pH and high bile salt and an ability to extend the lifespan of *Caenorhabditis elegans* infected with enterotoxigenic *Escherichia coli* (**ETEC**; *P* < 0.05). Thirty-two weaned piglets susceptible to ETEC F4 were randomly allocated to four treatments as follows: 1) non-challenged negative control group (**NNC**; basal diet and piglets gavaged with phosphate-buffered saline), 2) negative control group (**NC**; basal diet and piglets challenged with ETEC F4, 3 × 10^7^ CFU per pig), 3) positive control (**PC**; basal diet + 80 mg·kg^−1^ of avilamycin and piglets challenged with ETEC F4), and 4) probiotic candidate (PF9; control basal diet + 2.5 × 10^9^ CFU·kg^−1^ diet of *B. licheniformis* PF9 and piglets challenged with ETEC F4). The infection of ETEC F4 decreased average daily gain and gain:feed in the NC group when compared to the NNC group (*P* < 0.05). The inoculation of ETEC F4 induced severe diarrhea at 3 h postinoculum (**hpi**), 36, 40 hpi in the NC group when compared to the NNC group (*P* < 0.05). The supplementation of *B. licheniformis* PF9 significantly relieved diarrhea severity at 3 hpi when compared to the NC group (*P* < 0.05). The inoculation of ETEC F4 reduced duodenal, jejunal, and ileal villus height (**VH**) in the NC group when compared to the NNC group. A significant (*P* < 0.05) decrease was detected in the duodenal VH in the PC and NNC groups. Moreover, the NNC group had a reduced relative mRNA level of Na^+^-glucose cotransporter 1 (**SGLT1**) when compared to the NC group (*P* < 0.05). Compared to the NC and NNC groups, the supplementation of *B. licheniformis* PF9 increased the relative mRNA levels of aminopeptidase N, occludin, zonula occludens-1, and SGLT1 (*P* < 0.05). The supplementation of *B. licheniformis* PF9 also significantly increased the relative mRNA level of excitatory amino acid transporter 1 when compared to the NC group (*P* < 0.05). Piglets supplemented with *B. licheniformis* PF9 showed lower relative abundance of Bacteroidetes in the colon than piglets from the NNC group (*P* < 0.05). The NNC group had a higher relative abundance of Firmicutes in the ileum than all the challenged piglets (*P* < 0.05); however, a lower relative abundance of Proteobacteria in the ileum and colon was observed in the NC group (*P* < 0.05). This study provides evidence that *B. licheniformis* PF9 has the potential to improve the gut health of piglets under challenging conditions.

## Teaser

The pigs supplemented with *Bacillus licheniformis* PF9, which was tolerant to low pH and high bile salt, seemed to have better gut health under enterotoxigenic *E. coli* challenge conditions.

## INTRODUCTION

Postweaning diarrhea (**PWD**) is a common disease that causes a significant economic loss in the global swine industry (Rhouma et al., 2017). Enterotoxigenic *Escherichia coli* (**ETEC**) is the main infectious agent of PWD (Luppi, 2017). Antibiotic growth promoters (**AGPs**) have been commonly added to feed to improve animal growth performance and prevent infectious diseases such as PWD (Thoms, 2012). However, the use of AGPs in livestock production has been suggested to contribute to the development of antibiotics resistance, which threatens human health (Martin et al., 2015). The restriction of AGPs use in feed was enforced in European Union countries since 2006; in 2017, according to rules of the Food and Drug Administration of United States, animal producers required a veterinary feed directive for feed applications of antibiotics (Ekakoro et al., 2019); in Canada, prescriptions issued by veterinarians are needed for the use of all medically important antimicrobials (**MIA**) as defined by the World Health Organization, and growth promotion claims on the MIA product labels have been removed according to Health Canada regulations and policies implemented in 2018 (Lardé et al., 2021).

To overcome the potentially negative effects of removing AGPs, several AGPs alternatives, including pro- and prebiotics, zinc, copper, acidifiers, and plant extracts, have been tested (Liu et al., 2018). Among them, dietary supplementation of probiotics has been reported to have positive effects on improving gut health and immunity, nutrient digestibility, and suppressing pathogens, hence enhancing growth performance and preventing enteric diseases (Liao and Nyachoti, 2017). Various dietary probiotics have been studied in the past few decades. *Bacillus licheniformis* has been reported to have the function of secreting digestive enzymes to enhance nutrient digestion and absorption and antimicrobial peptides to inhibit the growth of *Brachyspira hyodysenteriae* and *Clostridium perfringens* (Rozs et al., 2001; Horng et al., 2019). Recently, our group reported that *B. licheniformis* PF9 was capable of reducing inflammation-related cytokines by blocking the NF-κB signaling pathways, while, significantly enhancing the intestinal porcine epithelial cell line integrity when infected with ETEC F4 (Li et al., 2022). It is hypothesized that dietary *B. licheniformis* PF9 will improve the gut health and growth performance of piglets. Therefore, the objective of the current report is to describe how *B. licheniformis* PF9 was selected and investigate its potential in application to improve the gut health and performance of weaned piglets including growth performance, digestive enzymes, gut morphology and integrity, nutrient transport, oxidative stress, innate immune responses, and gut microbiota after the ETEC F4 challenge.

## Materials and Methods

The pig trial and animal care protocols (F19-020, AC11516) were approved by the Animal Care Committee of the University of Manitoba. The piglets used in this trial were cared for under the guidelines of the Canadian Council on Animal Care (CCAC, 2009).

### *Bacillus licheniformis* PF9 and Its Culture Preparation

*Bacillus licheniformis* PF9 was recently shown through an in vitro study that it can improve barrier function and alleviate inflammatory responses against ETEC infection (Li et al., 2022). The bacterium was isolated from the feces of pigs raised on a free-range farm in Ontario, Canada, where antibiotics were not used in raising the pigs. It is heat resistant. The selection of a heat-resistant bacterium was conducted through a screening of multiple feces samples by suspending 0.5 g of pig feces in 4.5 mL of phosphate-buffered saline (**PBS**, pH 7.0) and heating the mixtures at 80 °C for 10 min followed by a series of dilution of each sample. The diluted samples were plated on the tryptic soy agar (**TSA**; Millipore Sigma, Oakville, ON, Canada) and incubated at 37 °C until colonies became visible. Finally, a single colony was selected for a pure bacterial culture.

To prepare a PF9 culture for the pig trial, a single colony of *B. licheniformis* PF9 from an agar plate was inoculated into 6 mL of tryptic soy broth (**TSB**; Millipore Sigma) medium in a 12-mL culture tube followed by the incubation at 37 °C for 16 h without shaking to get an initial culture. The initial culture was mixed with 250 mL of TSB in a 500-mL flask and incubated at 37 °C for 16 h without shaking. All the incubations were carried out aerobically. The culture (500 mL) with more than 99% of the cells in a vegetative form was evenly mixed with 50 kg of feed which included 2.5 × 10^9^ CFU·kg^−1^ of *B. licheniformis* PF9 in the diet.

### In Vitro Characterization of *B. licheniformis* PF9

To determine the species identity of isolate PF9, its 16S ribosomal RNA (**rRNA**) gene was sequenced and then blasted against the database sequences available on the National Center for Biotechnology Information website (http://www.ncbi.nlm.nih.gov). Further determination of isolate PF9 to be *B. licheniformis* or *B. paralicheniformis* was carried out using the method of particular molecular markers developed by Olajide et al. (2021). Both 16S rRNA gene sequencing and the marker detection to distinguish *B. licheniformis* from *B. paralicheniformis* were conducted by the Agriculture and Food Laboratory (Laboratory Service Division, University of Guelph, Guelph, Ontario, Canada), an ISO/IEC 17025 accredited laboratory (https://afl.uoguelph.ca/accreditations).

To determine the antimicrobial activity of *B. licheniformis* PF9, an inhibitory assay was carried out by measuring the culture optical density at 600 nm (**OD**_**600**_) of a target pathogen at 30-min intervals during an 18-h incubation at 37 °C with a Bioscreen C MBR (Oy Growth Curves Ab Ltd., Helsinki, Vuorimiehenkatu, Finland). The assay mixture (300 µL each) was constituted of 150 µL of TSB containing 1% inoculum of ETEC JG280 or *Salmonella* Typhimurium DT104 and 150 µL of filter-sterilized extracellular culture fluid of PF9. The control in the assay was the replacement of the extracellular culture fluid of PF9 with 150 µL of TSB without the bacterial inoculum, which was conducted in parallel.

To determine the tolerance of *B. licheniformis* PF9 to low pH and high bile salt, the isolate was tested based on the method previously reported (Yang et al., 2014) using the Bioscreen C MBR (Oy Growth Curves Ab Ltd.) to monitor the bacterial growth. Tolerance to low pH was evaluated by incubating bacterial cells in Lysogeny broth (**LB**; Millipore Sigma) at 37 °C, which had been treated with simulated gastric fluid (Wang et al., 2009) for 2 h with the pH values of 2.0 and 5.6, respectively. Tolerance to bile salt was investigated by incubating the isolate in the LB containing various concentrations (0%, 0.1%, 0.5%, 1.0%, and 1.5%) of bile salt (Catalog No. LP005, Oxoid, Nepean, ON, Canada) at 37 °C. The OD value of a sample was determined at a wavelength of 600 nm. The selected range of bile salts was based on the physiological concentration in the small intestine (Weinman et al., 1998).

### Culturing and Lifespan Assay of *Caenorhabditis elegans*

The SS104 strain of *C. elegans* harboring a temperature-sensitive allele of *glp-4* (*bn2*) was obtained from the Caenorhabditis Genetics Center (University of Minnesota, Minneapolis, MN, USA). *C. elegans* was maintained on nematode growth medium (**NGM**) plates seeded with *E. coli* OP50 (1 × 10^8^ CFU·mL^−1^) that was used as food for the nematode according to the procedures described by Breger et al. (2007). The ETEC strain JG280 is a hemolytic *E. coli* of serotype O149: K88 (F4), a porcine isolate possessing toxin genes and resistant to some antibiotics (Noamani et al., 2003).

The lifespan assays of *C. elegans* were conducted based on the previously published method (Zhou et al., 2014). Briefly, gravid adult worms were treated with sterile water (containing 0.5% NaClO solution and 0.5 M NaOH) to synchronize worms to the same age and stage. The eggs were released and isolated by centrifugation (1 min at 1,300 × *g*) and resuspended in M9 buffer, and then hatched for 16 h at 20 °C. The L1 larvae of *C. elegans* were transferred to NGM agar with *E. coli* OP50 (1 × 10^8^ CFU·mL^−1^) as food for the worms at 25 °C for 48–60 h until the worms reached the L4 stage. The worms collected from the NGM plate with M9 buffer were washed three times in the S medium, 18 to 26 worms were then transferred to each well containing 2 mL of S medium on a 24-well plate and incubated at 25 °C.

The treatments of the assay included 1) Control (treated with *E. coli* OP50 only), 2) ETEC infection (incubated with *E. coli* OP50 for 18 h followed by incubation of ETEC JG280 for 12 days), and 3) *B. licheniformis* PF9 pretreatment (incubated with *B. licheniformis* PF9 for 18 h followed by incubation of ETEC JG280 for 12 days). Each assay was designated as day 0 when the worms were fed *E. coli* OP50 or *B. licheniformis* PF9. The worms were collected after the 18-h incubation period via centrifugation and suspension and washed three times in the S medium. The assays were designated as day 1 when the washed worms were mixed with ETEC JG280 (2 × 10^8^ CFU mL^−1^) in a 24-well plate. The lifespan assay lasted for 12 days. The number of live worms was recorded daily, and the survival rate of worms was calculated by the formula: survival rate (%) = (live worms/total worms used) × 100. A worm was considered dead when it failed to respond to touching.

### ETEC F4 Culture Preparation

The ETEC F4 from −80 °C stock was streaked on the media of TSA to aerobically grow for 16 h at 37 °C. A single colony of ETEC F4 was used to inoculate 10 mL of TSB in a 50 mL sterile tube followed by aerobic incubation at 37 °C for 16 to 18 h with shaking (150 rpm). The culture was further subcultured by inoculating 10 mL TSB with 1% inoculum and incubated for 2.5 h at 37 °C with shaking (150 rpm) until its OD_600_ value reached 0.3. The ETEC F4 culture was diluted by PBS to obtain a cell suspension at approximately 1 × 10^7^ CFU·mL^−1^.

## Animal Experiment

### Selection of piglets

The piglets susceptible to ETEC F4 were selected for the ETEC challenge trial following the method described by Jensen et al. (2006). Briefly, the tails of piglets were collected 3 days after farrowing when docked. DNA was extracted according to previous publication (Truett et al., 2000). The mucin 4 (**MUC4**) gene was detected by a PCR assay. The assay mixture (25 µL) was composed of DNA polymerase (Thermo Fisher Scientific, Waltham, MA, USA), 200 µmol·L^−1^ of dNTP, 2 mmol·L^−1^ MgCl_2_, 400 µmol·L^−1^ of forward primer (5ʹ-GTGCCTTGGGTGAGAGGTTA-3ʹ), and reverse primer (5ʹ-CACTCTGCCGTTCTCTTTCC-3ʹ). Thermocycling was performed with initial denaturation at 95 °C for 5 min and subsequently by 35 cycles with each cycle having denaturation at 95 °C for 30 s, annealing at 65 °C for 30 s, and extension at 72 °C for 1 min. The PCR product size from the tail genomic DNA was 367 bp. The product (5 µL) was digested by XbaI (Thermo Fisher Scientific) for 5 min at 37 °C according to the protocols provided by the supplier. After digestion, the PCR products stained by SYBR Green (Invitrogen, Carlsbad, CA, USA) were electrophoresed on an agarose gel (2%) in a Tris-borate-EDTA buffer. The susceptible allele (S) was digested by FastDigest XbaI into two fragments with 151 and 216 bp, respectively, whereas the resistant allele (R) was indigestible. Similar body weight piglets contained both the SS-genotype (homozygote) and RS-genotype (heterozygote) were chosen for the experiment, as Matsumoto et al. (2020) revealed that RS-genotyped pigs showed no significant differences in fecal score, incidence of diarrhea, and diarrhea duration compared to SS-genotyped pigs.

### Experimental Design

Thirty-two weaned pigs susceptible to ETEC F4 (TN Tempo × TN70; half castrated male and half female piglets; average body weight of 8.15 ± 0.18 kg) were transferred to T.K. Cheung Centre for Animal Science Research from the Glenlea Swine Research Unit of the University of Manitoba at the age of 28 d (weaning at 21 d). All piglets were housed in individual pens in a temperature-controlled room with the room temperature kept at 29 ± 1 °C within the prechallenge period (0 to 7 d), and after that decreased by 1.5 °C during the postchallenge period (8 to 10 d). All piglets for this trial were randomly allocated to four treatments (eight replicates per treatment, half castrated male and half female). The basal diet (Table 1) based on corn–soy was made to meet or exceed the recommendations for 7 to 11 kg piglets from the NRC (2012). The treatments were designed as follows: 1) non-challenged negative control group (**NNC**; basal diet and piglets gavaged with PBS), 2) negative control group (**NC**; basal diet and piglets challenged with ETEC F4, 3 × 10^7^ CFU per pig), 3) positive control (**PC**; basal diet + 80 mg·kg^−1^ of avilamycin and piglets challenged with ETEC F4), and 4) probiotic candidate (PF9; control basal diet + 2.5 × 10^9^ CFU·kg^−1^ diet of *B. licheniformis* PF9 and piglets challenged with ETEC F4). The whole trial lasted for 10 days. Piglets had ad libitum access to feed and water throughout the whole trial. Individual piglet’s body weight and pen feed disappearance were recorded to calculate average daily feed intake (**ADFI**), average daily gain (**ADG**), and gain:feed within the prechallenge period and postchallenge period, respectively. For the ETEC F4 challenge, each piglet in the NC, PC, and PF9 groups was administered with 3 mL of ETEC F4 (1 × 10^7^ CFU·mL^−1^) on day 7 (Koo et al., 2020). The rectal temperature was measured by thermometer before the inoculation, 3, 24, and 48 h postinoculum (**hpi**). The diarrhea index score was recorded at 3, 8, 12, 16, 20, 24, 28, 32, 36, 40, and 48 hpi following the method of Marquardt et al. (1999).

**Table 1.** Composition and nutrient level of the basal diet^1^ (kg, as-fed basis)

Item	Basal diet
*Ingredients, %*	
Corn grain	48.4
Soybean meal	16.0
Fish meal	6.6
X-SOY600^2^ (600 g crude protein·kg^−1^)	11.0
Whey permeate	12.4
Soybean oil	1.5
Limestone	1.42
Salt	0.5
Biofos^3^	0.57
l-Lysine	0.28
Threonine	0.13
dl-Methionine	0.15
l-Tryptophan	0.05
Vitamin-mineral premix^4^	1.0
*Calculated nutrient levels, %*	
Crude protein	22.35
SID^5^ lysine	1.34
SID methionine	0.5
SID threonine	0.87
SID tryptophan	0.27
Metabolizable energy, kcal·kg^−1^	3,390
Net energy, kcal·kg^−1^	2,475

^1^The diet for PC was additionally added 80 mg·kg^−1^ of avilamycin (Surmax 100 Premix, avilamycin premix 100 g·kg^−1^, Elanco Canada Limited, Guelph, Ontario, Canada) based on the basal diet. The PF9 diet was added 2.5 × 10^9^ CFU·kg^−1^ of *B. licheniformis*.

^2^Soy protein concentration (CJ Selecta, Goiania, State of Goiás, Brazil).

^3^Monocalcium phosphate with 21% Ca and 17% P (Mosaic Co., Plymouth, MN, USA).

^4^Supplied per kilogram of diet as follows: 2,200 IU vitamin A, 1.5 mg vitamin B1, 0.02 mg vitamin B12, 0.2 mg biotin, 4 mg vitamin B2, 220 IU vitamin D3, 16 IU vitamin E, 0.5 mg vitamin K, 600 mg choline chloride, 30 mg niacin, 7 mg pyridoxine, 0.3 mg folic acid, 12 mg calcium pantothenate, 0.14 mg calcium iodate, 0.3 mg sodium selenite, 6 mg copper sulfate, 100 mg ferrous sulfate, 4 mg manganese oxide, and 100 mg zinc oxide.

^5^Standardized ileal digestible amino acids.

### Collection of Tissue and Digesta Samples

All piglets from this trial were anesthetized with ketamine–xylazine and euthanized by a captive bolt gun at the end of the trial (on day 11). First, the abdomen of the pig was quickly opened up, and the gut was quickly removed for sample collection. A 10 cm of jejunum (400 cm from the stomach-duodenum junction) was obtained, put in a tube containing cold Krebs ringer bicarbonate (**KRB**) buffer, and immediately transferred to the laboratory to perform the permeability assay using a Ussing chamber. The other 15 cm of the jejunum was collected and quickly frozen in liquid nitrogen prior to storage at −80 °C for follow-up analyses. A section of 3 cm each of the duodenum (8 cm from the stomach), mid-jejunum (4 m from the stomach-duodenum junction), and mid ileum (10 cm from the ileum-cecum junction) was removed and fixed in 10% formaldehyde solution for histological analysis of gut morphology. The digesta from the ileum (5 cm of ileum from the middle) and colon (2 cm of colon from the middle) were also obtained and quickly frozen in liquid nitrogen before storage at −80 °C for the follow-up analyses (Choi et al., 2020).

### In Vivo Gut Permeability

Each piglet received by oral gavage 10 mg in 5 mL PBS buffer of fluorescein isothiocyanate-dextran 70 kDa (**FITC-D70**; Sigma-Aldrich Co., St. Louis, MO, USA) on 3 d postinoculum. Four hours after gavage, blood samples from the jugular vein of each piglet were collected into vacutainer tubes (Becton Dickinson, Rutherford, NJ, USA) covered by aluminum foil for light blocking, and allowed to clot for 3 h at 22 °C. After centrifuging for 15 min at 750 × *g*, the obtained serum samples were stored at −80 °C for further analyses. The FITC-D70 content in the serum was measured by a Bio-Tek PowerWave HT Microplate Scanning Spectrophotometer (Bio-Tek Instruments Inc., Winooski, VT, USA) under the excitation wavelength of 485 nm and emission wavelength of 528 nm and calculated through the standard curve.

### Analysis of Transepithelial Electrical Resistance

Transepithelial electrical resistance (**TEER**) was determined as the electrophysiological property by a Ussing chamber (Physiologic Instruments Inc., San Diego, CA, USA) with voltage and current electrodes filled with KRB buffer and housed in 3% agar bridges. Five milliliters KRB buffer solution was filled in the mucosal chambers and the serosal chambers with 10 mmol·L^−1^d-mannitol and d-glucose, respectively. The chambers were kept at a temperature of 37 °C through a water-jacketed reservoir and continued to pump the 95% O_2_ and 5% CO_2_ mixed gas. The tissue without serosal and longitudinal muscle layers was mounted in Ussing chambers, equilibrated for 10 min, and then the TEER was recorded after mounting for 10 min (Choi et al., 2020).

### Gut Morphology

The gut tissue samples were embedded in paraffin after being fixed in a 10% formaldehyde solution, sliced into samples with 5 µm thickness, and then transferred onto glass slides. The dewaxed parts were immersed in xylene, 100% ethanol, and 95% ethanol for 5 min each for two cycles. After that, the gut tissues were left in Alcian blue at 22 °C for 15 min and washed with pure water for 2 min and in Schiff for 10 min followed by another washing in pure water for 10 min. Finally, hematoxylin was used for a second staining for 10 s, and then the samples were washed with water and dehydrated. Each slide was photographed and visualized using a digital camera (Lumenera Corp., Ottawa, ON, Canada) and microscope equipped with 10× lenses (Carl Zeiss Microscopy Deutschland GmbH, Göttingen, Germany) for quantification of Alcian blue/Periodic acid–Schiff staining. Fifteen villi and associated crypts of each sample were selected for measuring villus height (**VH**), crypt depth (**CD**), and VH:CD using a Infinity Analyze software (Lumenera Corp.; Choi et al., 2020).

### Analysis of Intestinal Digestive Enzyme Activities

Maximal enzyme activity (***V***_**max**_) of intestinal digestive enzymes in the mid-jejunum including sucrase, maltase, intestinal alkaline phosphatase (**IAP**), aminopeptidase N (**APN**), and maltase-glucoamylase (**MGA**) were all measured. Specifically, around 200 mg of frozen mid-jejunum tissues were homogenized on ice in a cold homogenizing buffer (phenylmethylsulfonyl fluoride and d-mannitol, pH 7.4) with a polytron homogenizer. The original tissue homogenate sample suspension was transferred into the tube to determine the protein content. The activities of sucrase and maltase were measured following the method written by Dahlqvist (1964). The activity of IAP was analyzed following the method of Hübscher and West (1965), and APN activity was measured using the procedure of Maroux et al. (1973). MGA activity was determined using the procedure of Lackeyram et al. (2012).

### Assays of Total Glutathione, Glutathione/Oxidized Glutathione, and Antioxidant Capacity

Total antioxidant capacity (**TAC**) in the mid-jejunum was analyzed using the Total Antioxidant Capacity Kits of Colorimetric Microplate Assay (Oxford Biomedical Research Inc., Oxford, MI, USA; Yang, 2011). Specifically, about 200 mg of mid-jejunal tissues were homogenized with 1 mL of cold PBS for 30 s on ice and centrifuged for 12 min at 3,600 × *g* at 4 °C. Firstly, 25 µL supernatant was used for protein level analysis. Secondly, the TAC in an aliquot of supernatant was determined by all antioxidants’ capacity to convert Cu^2+^ to Cu^+^ following the manufacturer’s protocol. Cu^+^ was complexed with bathocuproine to create a stable form which was detected by Bio-Tek PowerWave HT Microplate Scanning Spectrophotometer (Bio-Tek Instruments Inc.) with a 96-well plate reader at a wavelength of 450 nm. The values were based on the standard curve (Choi et al., 2020).

Glutathione (**GSH**) and oxidized glutathione (**GSSG**) levels of the mid-jejunum were analyzed using the detection kit for glutathione colorimetric according to the manufacturer’s instructions (Invitrogen). Specifically, around 30 mg of tissues were homogenized for 30 s on ice in 750 µL of ice-cold PBS followed by centrifugation for 10 min at 3,600 × *g* at 4 °C. After determining the protein concentration, 5-sulfosalicylic acid dihydrate used for precipitating protein was added into the supernatant followed by another centrifugation for 10 min at 3,600 × *g* at 4 °C. The protein level of all homogenized tissues was measured by a BCA Protein Assay Kit (Thermo Fisher Scientific). The total GSH and GSSG levels were then measured using this equation: Reduced GSH = Total GSH − 2 × GSSG (Choi et al., 2020).

### RNA Extraction and Gene Expression Analysis

Total RNA in 50 mg mid-jejunal tissues was extracted by the total RNA isolation kit (Thermo Fisher Scientific Inc.). The RNA concentration and OD_260_:OD_280_ ratios were measured by a Nanodrop spectrophotometer (Thermo Fisher Scientific Inc., Ottawa, ON, Canada). One microgram of RNA was used for synthesizing cDNA using an iScript cDNA Synthesis Kit (Bio-Rad Laboratories, Mississauga, ON, Canada) following the protocol provided by the manufacturer. Primer-Blast was used for designing the primers (Table 2) and synthesized by Integrated DNA Technologies, Inc. (Coralville, IA, USA). A total volume of 20 μL containing 1 μL cDNA, 300 nmol·L^−1^ of each forward and reverse primers and 10 μL SYBR Green supermix (Bio-Rad, Ontario, Canada) was used for Real-time PCR assays, which were performed on a Real-Time PCR Detection System (Bio-Rad Laboratories, Ontario; Omonijo et al., 2019). All reaction conditions were denaturation at 95 °C for 3 min, 40 cycles of 20 s at 95 °C, 30 s at 60 °C, and then 30 s at 72 °C. The target mRNA abundance was normalized with GAPDH and relative mRNA levels were determined by the 2^−ΔΔ^CT method (Livak and Schmittgen, 2001). The values of the threshold cycle (**Ct**) were acquired when targeted genes were amplified above a threshold of 30 fluorescence units. Duplication of each gene was performed for each sample (Choi et al., 2020).

**Table 2.** Primer sequences of ETEC F4 receptor, nutrient transporters, tight junction proteins, digestive enzymes, and inflammatory cytokines of piglets^1^

Genes	Amplicon, bp	Sequence (5ʹ to 3ʹ)	References
MUC2	90	CCAGGTCGAGTACATCCTGC GTGCTGACCATGGCCCC	Choi et al. (2020)
MUC4	367	GTGCCTTGGGTGAGAGGTTA CACTCTGCCGTTCTCTTTCC	Jensen et al. (2006)
ZO-1	200	GATCCTGACCCGGTGTCTGA TTGGTGGGTTTGGTGGGTT	Omonijo et al. (2018)
OCLN	93	CTGTGGATGTCCTGCGTGT GGTTGCTTGCAAAGTGGTGTT	Lee and Kang (2017)
CLDN3	123	CTACGACCGCAAGGACTACG TAGCATCTGGGTGGACTGGT	Omonijo et al. (2018)
B^0^AT1	102	AGGCCCAGTACATGCTCAC CATAAATGCCCCTCCACCGT	Yang et al. (2016)
SGLT1	153	GGCTGGACGAAGTATGGTGT GAGCTGGATGAGGTTCCAAA	Yang et al. (2011)
PepT1	143	ATCGCCATACCCTTCTG TTCCCATCCATCGTGACATT	Omonijo et al. (2018)
ASCT2	206	GCCAGCAAGATTGTGGAGAT GAGCTGGATGAGGTTCCAAA	Yang et al. (2016)
EAAC1	168	CCAAGGTCCAGGTTTTGGGT GGGCAGCAACACCTGTAATC	Omonijo et al. (2018)
MGA	118	GCCCCTTCTGCATGAGTTCT CGTCACTTTCTCTGCACCCT	Choi et al. (2020)
APN	114	GGACGATTGGGTCTTGCTGA GGGATGACCGACAGGTTTGT	Choi et al. (2020)
IL-1β	91	TGGCTAACTACGGTGACAACA CCAAGGTCCAGGTTTTGGGT	Choi et al. (2020)
IL-8	126	CACCTGTCTGTCCACGTTGT AGAGGTCTGCCTGGACCCCA	Omonijo et al. (2018)
IL-10	220	CATCCACTTCCCAACCAGCC CTCCCCATCACTCTCTGCCTTC	Lee and Kang (2017)
TLR2	109	ACATGAAGATGATGTGGGCC TAGGAGTCCTGCTCACTGTA	Tohno et al. (2005)
TLR5	86	GTTCTTTATCCGGGTGACTT AATAAGTCAGGATCGGGAGA	Choi et al. (2020)
TLR7	107	GCTGTTCCCACTGTTTTGCC GAGCTGGATGAGGTTCCAAA	Choi et al. (2020)

^1^MUC2, mucin 2; MUC4, mucin 4; OCLN, occludin; ZO-1, zonula occludens-1; CLDN3, claudin 3; EAAC1, excitatory amino acid transporter 1; ASCT2, neutral amino acid transporter 2; B^0^AT1, neutral amino acid transporter; PepT1, peptide transporter 1; SGLT1, Na^+^-glucose cotransporter 1; MGA, maltase-glucoamylase; APN, aminopeptidase N; IL-1β, interleukin-1β; IL-8, interleukin-8; IL-10, interleukin-10; TLR2, toll-like receptor 2; TLR5, toll-like receptor 5; TLR7, toll-like receptor 7.

### Microbiota Analysis

The PCR amplification and 16S rRNA sequencing were performed by Genome Quebec. Bacterial DNA from ileal and colonic digesta was extracted using a Fast DNA Stool Mini Kit (Qiagen, Hilden, Germany) according to the manufacturer’s protocol. Extracted DNA from the digesta was amplified by PCR, targeting the V3-V4 hypervariable region of the 16S rRNA gene with a forward primer 341 F (5ʹ-CCTACGGGNGGCWGCAG-3ʹ) and a reverse primer 805 R (5ʹ-GACTACHVGGGTATCTAATCC-3ʹ; Caporaso et al., 2012).

The PCR reaction system was 25 µL, containing 1× reaction buffer (Q5), 5% DMSO, l µL DNA template, 0.2 mM dNTP, 0.5 U Q5 DNA polymerase, and 0.6 µM of forward and reverse primers. The PCR amplifications were performed according to the following procedures: 30 s of 98 °C denaturation, followed by 25 cycles of 10 s at 98 °C, annealing at 60 °C for 15 s, and elongation at 72 °C for 30 s, with a final extension at 72 °C for 2 min. After verification of the amplificons on 2% agarose gel, the barcoding step was conducted with a total volume of 20 µL, containing 1× reaction buffer, 5% DMSO, 0.2 mM dNTP, 1.8 mM Mg^2+^, 0.2 µM primers, 0.5 U polymerase, and 1 µL DNA template. Initially, the PCR program started with 95 °C for 10 min; 95 °C for 15 s, 60 °C for 30 s, and 72 °C for 1 min, repeat for 15 cycles; 72 °C for 3 min. The verification of barcode incorporation for each sample was then on 2% agarose gel. Each amplicon was quantified with the QuantiT PicoGreen dsDNA Assay Kit (Thermo Fisher Scientific). The library was then generated by pooling the same quantity of each amplicon and cleaning-up the pool with sparQ PureMag Beads (Quantabio, Beverly, MA, USA). Afterward, the library was quantified using Kapa Illumina GA with Revised Primers-SYBR Fast Universal kit (Sigma-Aldrich Canada Co., Oakville, ON, Canada). The average fragment size was determined using a LabChip GX (PerkinElmer, Waltham, MA, USA) instrument. Before sequencing, 5% of the PhiX control library was added to the amplicon pool (loaded at a final concentration of 10 pM). The PhiX control library helps to improve the unbalanced base composition of the flowcell. Illumina MiSeq sequencing was carried out by Genome Quebec. Sequences were inputted into Quantitative Insights Into Microbial Ecology 2 (QIIME2; version 2021.8) for bioinformatics analysis (Caporaso et al., 2010; Bolyen et al., 2019).

Plugin q2-dada2 was applied for features construction and quality control (Callahan et al., 2016). Taxonomic classification was then conducted by the feature-classifier plugin (Quast et al., 2013; Bokulich et al., 2018).

### Statistical Analyses

Survival curves for *C. elegans* were compared using the Kaplan-Meier survival analysis of SAS (V.3.8; SAS Institute, Cary, NC, USA) followed by a log-rank test. One-way analysis of variance (**ANOVA**) and the Tukey’s multiple comparisons were used to test for significant differences between the means. Tolerance to low pH and high bile salt data were analyzed by the SAS GLM procedure and Tukey multiple comparisons. Means with *P* < 0.05 were considered a significant difference.

PROC MIXED of SAS with each piglet as the experimental unit in a randomized complete block design were used for analyzing the data. The model included sex (barrows and gilts) and treatment as the main effects and the interaction of sex by treatment. The least-square means with the option of Tukey-adjusted PDIFF was applied to split and calculate the treatment mean value. The NC group was compared with either the NNC group by preplanned contrasts to determine the effects of ETEC F4, or the PC group or the PF9 group to determine the avilamycin or the *B. licheniformis* effects, respectively. Results shown in tables are indicated as LSMEANS and pooled SEM, while results shown in figures are indicated as mean ± SEM. A *P*-value of <0.05 was considered as a significant difference.

Statistical analysis and data visualization for ileal and colonic microbiota were performed by the R program (version 4.0.2). Chao1, Shannon, and Simpson alpha diversity indices and relative abundance were performed by the phyloseq package. Ggplot2 package in the R program was applied to visualize the relative abundance, which aggregated at different taxonomical levels. Significant relative abundance was observed by the Kruskal–Wallis rank sum test. The conover test with the agricolae package was used for multiple pairwise comparison. Beta diversity was performed by the Bray–Curtis dissimilarity.

## RESULTS

### Species Identification of Isolate PF9 and Its Tolerance to Low pH and High Bile Salt

According to the final test report from the Agriculture and Food Laboratory, University of Guelph, sequencing of the 16S rRNA gene (1,485 bp) of isolate PF9 revealed >99.8% similarity to *B. licheniformis* or *B. paralicheniformis* compared with known 16S rRNA gene sequences in the NCBI GenBank database. In contrast to a *B. paralicheniformis* strain that served as a positive control in the assay, isolate PF9 showed no amplicons of *fenC* and *fenD*, the two markers from the fengycin operon of *B. paralicheniformis*, confirming that the isolate PF9 belongs to *B. licheniformis*. In the antimicrobial assays, the isolate partially inhibited the growth of ETEC JG280 and *Salmonella* Typhimurium DT104 (18%–30%).

**Figure 1.**
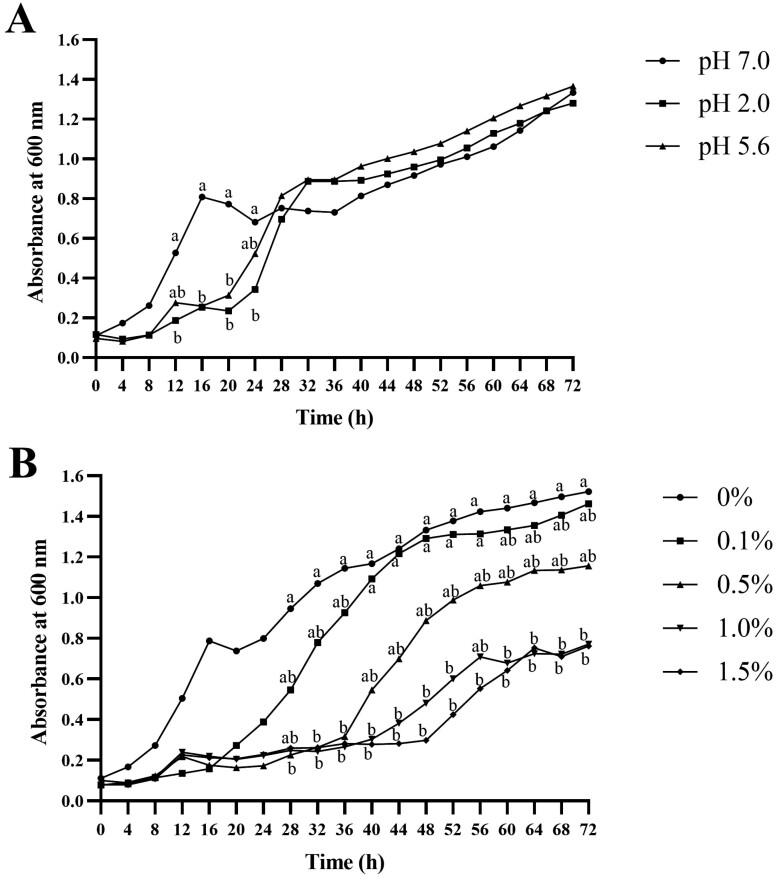
Tolerance of *B. licheniformis* PF9 to low pH (A) and high bile salt (B). Different letters represent a signiffcant difference (*P* < 0.05).

In the tolerance assay to low pH, *B. licheniformis* PF9 had similar optical OD_600_ values after 28 h growth at pH 7.0 regardless of the culture being pretreated at pH 2.0 or 5.6, indicating its tolerance to low pH (Fig. 1A). In the tolerance assay to high bile salt, *B. licheniformis* PF9 at 0.1% (v/v) concentration of bile salt showed similar growth with the control (0%) group (Fig. 1B). At 0.5% (v/v), the isolate had poorer growth than the control group before 36 h incubation. However, after 72 h, the growth of the isolate reached more than 1.0 at OD_600_ without a significant difference (*P* > 0.05) compared to the control (0%). At 1.0% and 1.5% (v/v), the growth of the isolate was suppressed until 48 h incubation. After 72 h, the same cultures had about half of the OD_600_ values of the control (*P* < 0.05). These results suggest that *B. licheniformis* PF9 has a certain degree of tolerance to bile salt.

### Protection to *C. elegans* Infected With ETEC F4

**Figure 2.**
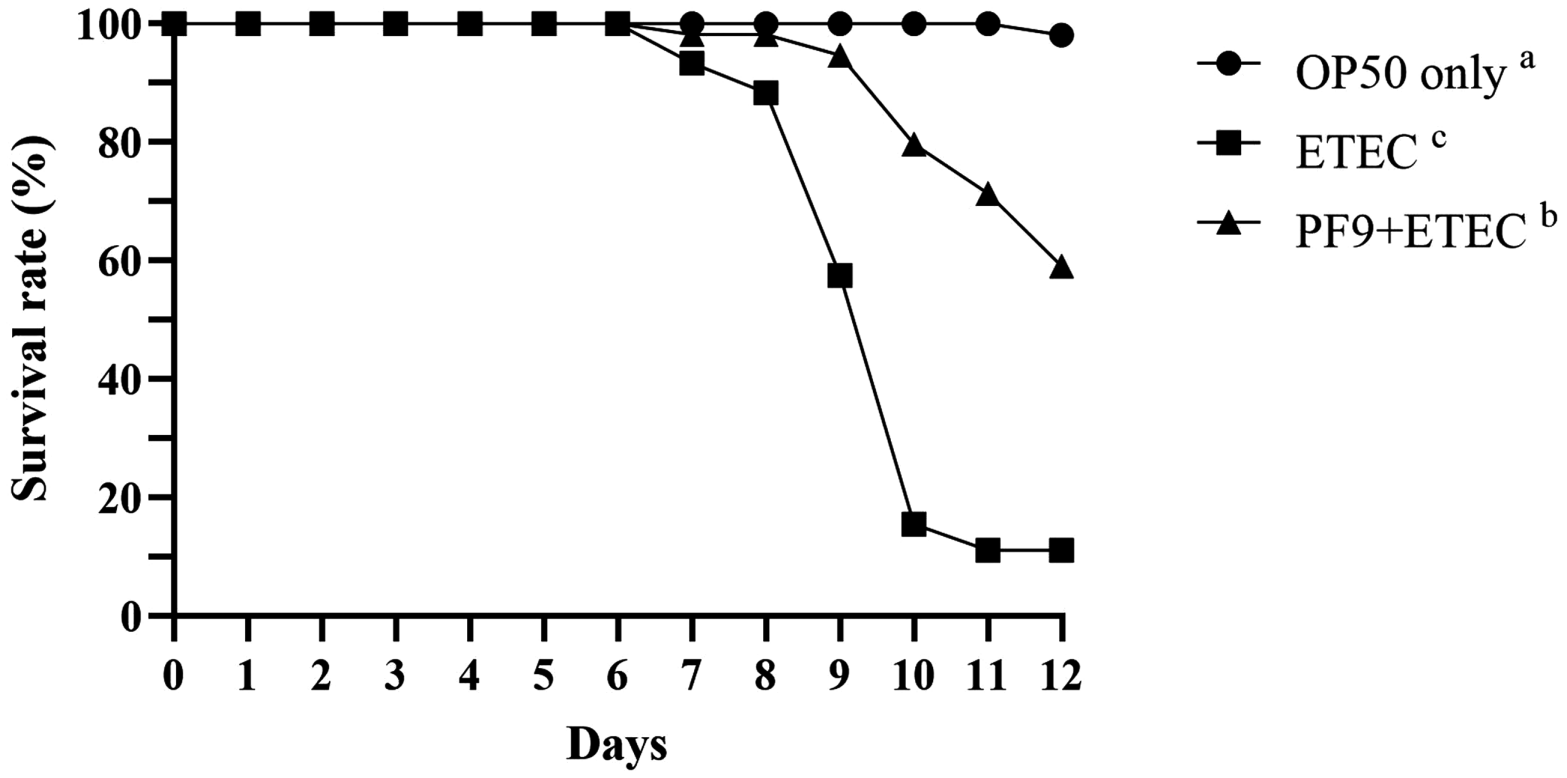
Lifespan of *C. elegans* with or without the pretreatment of *B. licheniformis* isolate PF9 after infection with enterotoxigenic *Escherichia coli* (ETEC). Treatments: ●, treated with *E. coli* OP50 only; ■, treated with ETEC JG280; ▲, treated with *B. licheniformis* isolate PF9 and then ETEC JG280. All the groups showing different letters were significantly different (*P* < 0.05) in their survival curves.

Figure 2 shows the effects of *B. licheniformis* PF9 on the lifespan of *C. elegans* infected with ETEC JG280. The lifespan of worms infected with ETEC JG280 was dramatically decreased after 8 d of incubation. However, the nematode pretreated with *B. licheniformis* PF9 had a significantly extended lifespan (*P* < 0.05) compared to those infected with ETEC JG280 only. On day 12, only about 10% of the worms survived the ETEC infection, while the survival rate was increased to nearly 60% by the *B. licheniformis* PF9 pretreatment.

### Growth Performance, Diarrhea Index Score, and Rectal Temperature

**Figure 3.**
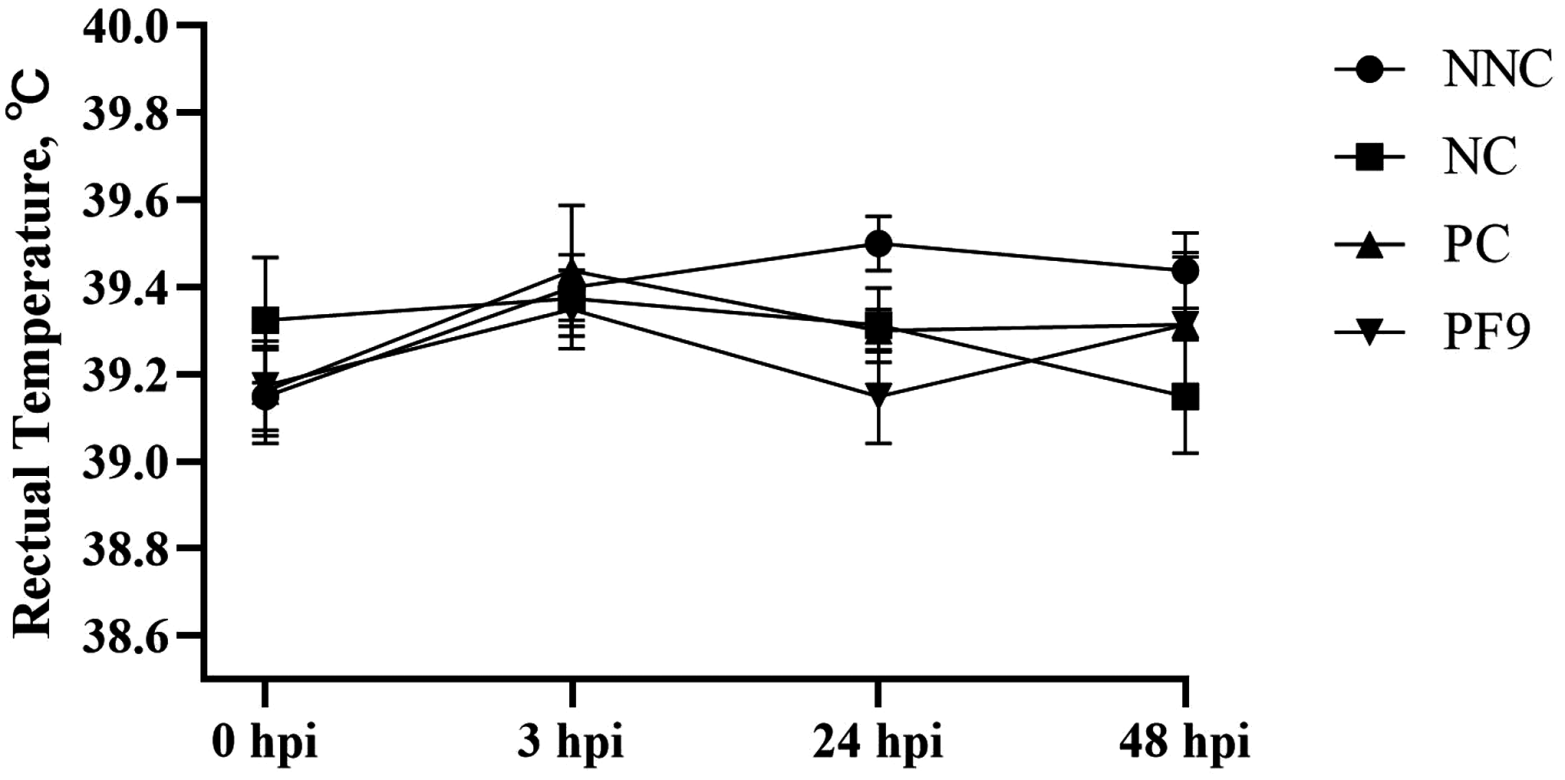
Effects of *B. licheniformis* PF9 on rectal temperature in piglets. Rectal temperature was measured in the non-challenged negative control (NNC) group with basal diet and piglets gavaged with PBS; negative control (NC) group with basal diet and piglets challenged with enterotoxigenic *E. coli* (ETEC) F4; positive control (PC) group with basal diet + 80 mg·kg^−1^ of avilamycin and piglets challenged with ETEC F4; PF9 group with basal diet + 2.5 × 10^9^ CFU·kg^−1^ diet of *B. licheniformis* and piglets challenged with ETEC F4. Each value is represented by the mean ± SEM.

**Figure 4.**
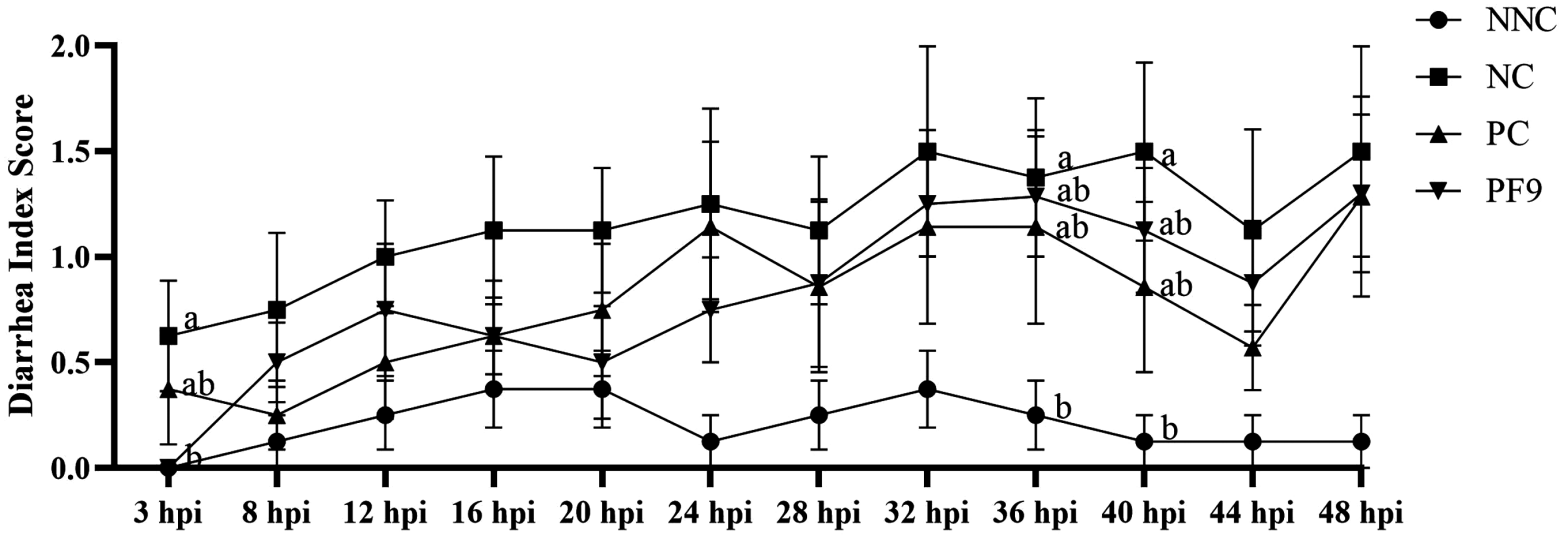
Effects of *B. licheniformis* PF9 on diarrhea index score in weaned piglets. The diarrhea index score was measured in the non-challenged negative control (NNC) group with basal diet and piglets gavaged with PBS; negative control (NC) group with basal diet and piglets challenged with enterotoxigenic *E. coli* (ETEC) F4; positive control (PC) group with basal diet + 80 mg·kg^−1^ of avilamycin and piglets challenged with ETEC F4; PF9 group with basal diet + 2.5 × 10^9^ CFU·kg^−1^ diet of *B. licheniformis* and piglets challenged with ETEC F4. Diarrhea index score 0 represents normal feces; 1 represents soft feces; 2 represents mild diarrhea; and 3 represents severe diarrhea. Each value is represented by the mean ± SEM. Different letters shown in the bars indicate significant difference (*P* < 0.05).

The statistical analysis demonstrated no significant interactions (*P* > 0.05) of sex and treatments for the variables studied (data not shown). Therefore, both male and female pigs were combined for the following analyses. There was no significant difference (*P* > 0.05) in the ADG, ADFI, and gain:feed found among all groups during the prechallenge period (0 to 7 d) and whole period (0 to 10 d; Table 3). The ADFI among all treatment groups during the postchallenge period did not show any significant differences (*P* > 0.05). Compared to the NNC group, the inoculation of ETEC F4 significantly decreased ADG and gain:feed in the NC group (*P* < 0.05). As shown in Fig. 3, no significant difference (*P* > 0.05) was found in the rectal temperature observed among all treatments during 48 hpi. Compared to the NNC group, the ETEC F4 challenge significantly (*P* < 0.05) increased the severity of diarrhea at 3, 36, and 40 hpi in the NC group. The supplementation of *B. licheniformis* significantly (*P* < 0.05) reduced diarrhea score at 3 hpi compared to the NC group (Fig. 4).

**Table 3.** Effects of *B. licheniformis* PF9 on the growth performance of weaned piglets during the prechallenge period (0 to 7 d), postchallenge period (8 to 10 d), and whole period

Items^1^		Challenged groups			SEM	*P* value			
	NNC	NC	PC	PF9		TRT	NNC vs. NC	NC vs. PC	NC vs. PF9
*Prechallenge period*									
ADG, g·d^−1^	264.50	270.09	241.38	274.39	17.57	0.91	0.99	0.93	0.99
ADFI, g·d^−1^	348.03	346.50	347.74	351.55	19.32	0.99	1.00	1.00	0.99
Gain:Feed	0.767	0.759	0.747	0.775	0.019	0.96	1.00	0.99	0.99
*Postchallenge period*									
ADG, g·d^−1^	389.42^a^	208.40^b^	327.47^ab^	297.83^ab^	20.30	0.01	0.01	0.06	0.29
ADFI, g·d^−1^	607.00	498.92	559.80	572.44	20.35	0.20	0.35	0.42	0.20
Gain:Feed	0.709^a^	0.481^b^	0.556^ab^	0.576^ab^	0.026	0.01	0.01	0.08	0.27
*Whole period*									
ADG, g·d^−1^	331.26	231.96	260.68	295.15	17.26	0.17	0.19	0.89	0.32
ADFI, g·d^−1^	468.97	417.91	365.04	404.81	17.71	0.36	0.76	0.67	0.99
Gain:Feed	0.749	0.573	0.600	0.721	0.027	0.04	0.10	0.94	0.21

^1^NNC, non-challenged negative control group with basal diet and piglets gavaged with PBS; NC, negative control group with basal diet and piglets challenged with ETEC F4; PC, positive control group with basal diet + 80 mg·kg^−1^ of avilamycin and piglets challenged with ETEC F4; PF9, isolate PF9 group with basal diet + 2.5 × 10^9^ CFU·kg^−1^ diet of *B. licheniformis* and piglets challenged with ETEC F4; ADG, average daily gain; ADFI, average daily feed intake.

^a,b^Values within a row with different superscripts differ significantly at *P* < 0.05.

### Gut Morphology

The inoculation of ETEC F4 significantly (*P* < 0.05) decreased duodenal, jejunal, and ileal VH in the NC group compared to the NNC group, and there was a significant decrease (*P* < 0.05) in duodenal VH between the PC and NNC group (Table 4). However, there was no significant difference (*P* > 0.05) in all small intestinal CD and VH:CD among all treatments.

**Table 4.** Effects of *B. licheniformis* PF9 on gut morphology with villus height (VH, μm), crypt depth (CD, μm), and VH:CD in the duodenum, jejunum, and ileum of weaned piglets

Items^1^		Challenged groups			SEM	*P* value			
	NNC	NC	PC	PF9		TRT	NNC vs. NC	NC vs. PC	NC vs. PF9
*Duodenum*									
VH	555^a^	467^b^	472^b^	514^ab^	10.33	0.01	0.04	0.99	0.25
CD	158	156	157	158	2.54	0.99	0.99	0.99	0.99
VH:CD	3.32	3.03	3.00	3.18	0.06	0.21	0.42	0.99	0.86
*Jejunum*									
VH	519^a^	445^b^	465^ab^	470^ab^	6.72	0.02	0.01	0.65	0.34
CD	148	145	144	147	1.93	0.92	0.98	0.99	0.99
VH:CD	3.20	3.14	3.26	3.26	0.05	0.81	0.97	0.86	0.82
*Ileum*									
VH	411^a^	361^b^	386^ab^	401^ab^	5.69	0.03	0.03	0.44	0.11
CD	140	149	140	143	2.15	0.49	0.54	0.51	0.74
VH:CD	3.02	2.66	2.71	2.79	0.05	0.08	0.11	0.99	0.77

^1^NNC, non-challenged negative control group with basal diet and piglets gavaged with PBS; NC, negative control group with basal diet and piglets challenged with ETEC F4; PC, positive control group with basal diet + 80 mg·kg^−1^ of avilamycin and piglets challenged with ETEC F4; PF9, isolate PF9 group with basal diet + 2.5 × 10^9^ CFU·kg^−1^ diet of *B. licheniformis* and piglets challenged with ETEC F4.

^a,b^Values within a row with different superscripts differ significantly at *P* < 0.05.

### Gut Permeability

As shown in Table 5, there was no significant difference (*P* > 0.05) observed in the TEER measured by the Ussing chamber and in vivo gut permeability among all treatments.

**Table 5.** Effects of *B. licheniformis* PF9 on electrophysiological properties with transepithelial electrical resistance (TEER, Ω·cm^2^) in jejunum mounted in Ussing chambers and flux of fluorescein isothiocyanate-dextran 70 kDa (FITC-D70, μg·m^−1^) in weaned piglets

Items^1^		Challenged groups			SEM	*P* value			
	NNC	NC	PC	PF9		TRT	NNC vs. NC	NC vs. PC	NC vs. PF9
*Ex vivo*									
TEER	51.62	52.13	56.42	55.64	3.16	0.91	0.99	0.97	0.99
*In vivo*									
FITC-D70 flux	0.77	0.92	0.87	0.83	0.03	0.13	0.10	0.92	0.49

^1^NNC, non-challenged negative control group with basal diet and piglets gavaged with PBS; NC, negative control group with basal diet and piglets challenged with ETEC F4; PC, positive control group with basal diet + 80 mg·kg^−1^ of avilamycin and piglets challenged with ETEC F4; PF9, isolate PF9 group with basal diet + 2.5 × 10^9^ CFU·kg^−1^ diet of *B. licheniformis* and challenged with ETEC F4.

### Intestinal Digestive Enzyme Activities, Total GSH, GSH/GSSG, and TAC

There was no significant difference (*P* > 0.05) in the *V*_max_ values of APN, IAP, MGA, sucrase, and maltase among all treatments (Table 6). No significant difference (*P* > 0.05) was found in the GSH, GSSG, reduced GSH, reduced GSH:GSSG ratio, and TAC among all treatments (Table 6).

**Table 6.** Effects of *B. licheniformis* PF9 on the digestive enzymes’ activities (nmol·L^−1^·mg protein^−1^·min^−1^), the total glutathione (GSH, nmol·L^−1^·mg protein^−1^), oxidized glutathione (GSSG, nmol·L^−1^·mg protein^−1^), reduced GSH:GSSG, and total antioxidant capacity (TAC, mmol·L^−1^·mg protein^−1^) in the mid-jejunum of weaned piglets

Items^1^		Challenged groups			SEM	*P* value			
	NNC	NC	PC	PF9		TRT	NNC vs. NC	NC vs. PC	NC vs. PF9
IAP	0.35	0.49	0.35	0.32	0.04	0.34	0.60	0.38	0.28
APN	0.15	0.13	0.14	0.14	0.01	0.98	0.98	0.99	0.99
Maltase	105.09	101.88	109.86	117.94	10.32	0.96	0.99	0.99	0.96
MGA	5.24	5.98	7.61	7.95	0.99	0.75	0.99	0.94	0.94
Sucrase	9.73	10.67	11.71	12.93	1.15	0.80	0.96	0.99	0.87
Total GSH	2.62	2.19	2.26	2.54	0.28	0.94	0.97	0.99	0.95
GSSG	0.51	0.39	0.37	0.54	0.08	0.88	0.96	0.99	0.92
Reduced GSH^2^	1.68	1.56	1.62	1.33	0.17	0.73	0.99	0.99	0.84
Reduced GSH:GSSG	3.67	4.77	4.02	3.66	0.68	0.93	0.97	0.96	0.93
TAC	69.06	64.04	68.91	66.24	2.47	0.94	0.96	0.96	0.99

^1^IAP, intestinal alkaline phosphatase; APN, aminopeptidase N; MGA, maltase-glucoamylase; NNC, non-challenged negative control group with basal diet and piglets gavaged with PBS; NC, negative control group with basal diet and piglets challenged with ETEC F4; PC, positive control group with basal diet + 80 mg·kg^−1^ of avilamycin and piglets challenged with ETEC F4; PF9, isolate PF9 group with basal diet + 2.5 × 10^9^ CFU·kg^−1^ diet of *B. licheniformis* and piglets challenged with ETEC F4.

^2^Reduced GSH = Total GSH − 2 × GSSG.

### Relative mRNA Abundance in the Jejunum

The relative mRNA levels of genes in the mid-jejunum related to gut barrier function, nutrient transportation, digestive enzymes, and immunity were determined by Real-time PCR assays. As shown in Table 7, no significant difference (*P* > 0.05) was observed in the relative mRNA levels of claudin 3, MUC2, maltase-glucoamylase (**MGA**), peptide transporter 1, neutral amino acid transporter 2, neutral amino acid transporter, interleukin-1β (**IL-1β**), **IL-6**, **IL-8**, **IL-10**, toll-like receptor 5 (**TLR5**), and **TLR7** among all treatments. Compared to the NNC group, the inoculation of ETEC F4 significantly (*P* < 0.05) decreased the relative mRNA levels of Na^+^-glucose cotransporter 1 (**SGLT1**) in the NC group. When compared to the NC and NNC groups, the group supplemented with *B. licheniformis* PF9 significantly (*P* < 0.05) increased the relative mRNA levels of SGLT1, occludin (**OCLN**), zonula occludens-1 (**ZO-1**), and APN. Moreover, the supplementation of *B. licheniformis* significantly (*P* < 0.05) increased the relative mRNA level of excitatory amino acid transporter 1 (**EAAC1**) when compared to the NC group.

**Table 7.** Effects of *B. licheniformis* PF9 on the relative mRNA levels of genes related to gut barrier function, nutrient transportation, immunity, and digestive enzymes in the mid-jejunum of weaned piglets^1^

Items^1^		Challenged groups			SEM	*P* value			
	NNC	NC	PC	PF9		TRT	NNC vs. NC	NC vs. PC	NC vs. PF9
*Gut barrier function*									
ZO-1	1.08^b^	1.02^b^	1.64^ab^	2.38^a^	0.20	<0.01	0.99	0.77	<0.01
CLDN3	1.01	0.84	1.05	1.31	0.09	0.47	0.75	0.55	0.51
OCLN	1.01^b^	1.08^b^	1.71^ab^	2.76^a^	0.21	<0.01	0.98	0.49	<0.01
MUC2	1.02	0.97	1.00	0.85	0.05	0.83	0.99	0.99	0.89
*Nutrient transportation*									
SGLT1	1.00^b^	0.57^c^	0.76^abc^	1.72^a^	0.13	<0.01	0.04	0.92	<0.01
PepT1	1.04	0.92	1.05	1.37	0.09	0.33	0.89	0.96	0.28
B^0^AT1	1.00	0.74	0.90	1.25	0.12	0.53	0.57	0.96	0.61
EAAC1	1.08^ab^	0.42^b^	0.73^ab^	1.23^a^	0.13	0.03	0.09	0.83	0.04
ASCT2	1.05	0.91	1.41	1.12	0.10	0.35	0.95	0.32	0.87
*Digestive enzymes*									
MGA	1.01	0.75	0.74	1.33	0.10	0.08	0.32	1.00	0.07
APN	1.03^b^	0.96^b^	1.23^ab^	1.90^a^	0.11	<0.01	0.99	0.77	<0.01
*Immunity*									
IL-6	1.08	1.04	0.91	1.05	0.11	0.98	0.99	0.99	1.00
IL-8	1.04	1.09	0.82	0.76	0.08	0.12	0.99	0.63	0.12
IL-10	1.09	0.82	1.26	1.32	0.15	0.54	0.92	0.82	0.50
IL-1β	1.03	1.11	0.51	0.51	0.06	0.12	0.99	0.38	0.37
TLR5	1.05	0.97	1.29	1.68	0.13	0.24	0.98	0.83	0.21
TLR7	1.07	0.98	1.03	1.01	0.10	0.99	0.99	0.99	0.99

^1^MUC2, mucin 2; ZO-1, zonula occludens-1; OCLN, occludin; CLDN3, claudin 3; PepT1, peptide transporter 1; SGLT1, Na^+^-glucose cotransporter 1; ASCT2, neutral amino acid transporter 2; B^0^AT1, neutral amino acid transporter; EAAC1, excitatory amino acid transporter 1; MGA, maltase- glucoamylase; APN, aminopeptidase N; IL-1β, interleukin-1β; IL-6, interleukin-6; IL-8, interleukin-8; IL-10, interleukin-10; TLR5, toll-like receptor 5; TLR7, toll-like receptor 7.

^a,b^Values within a row with different superscripts differ significantly at *P* < 0.05.

### Gut Microbiota

**Figure 5.**
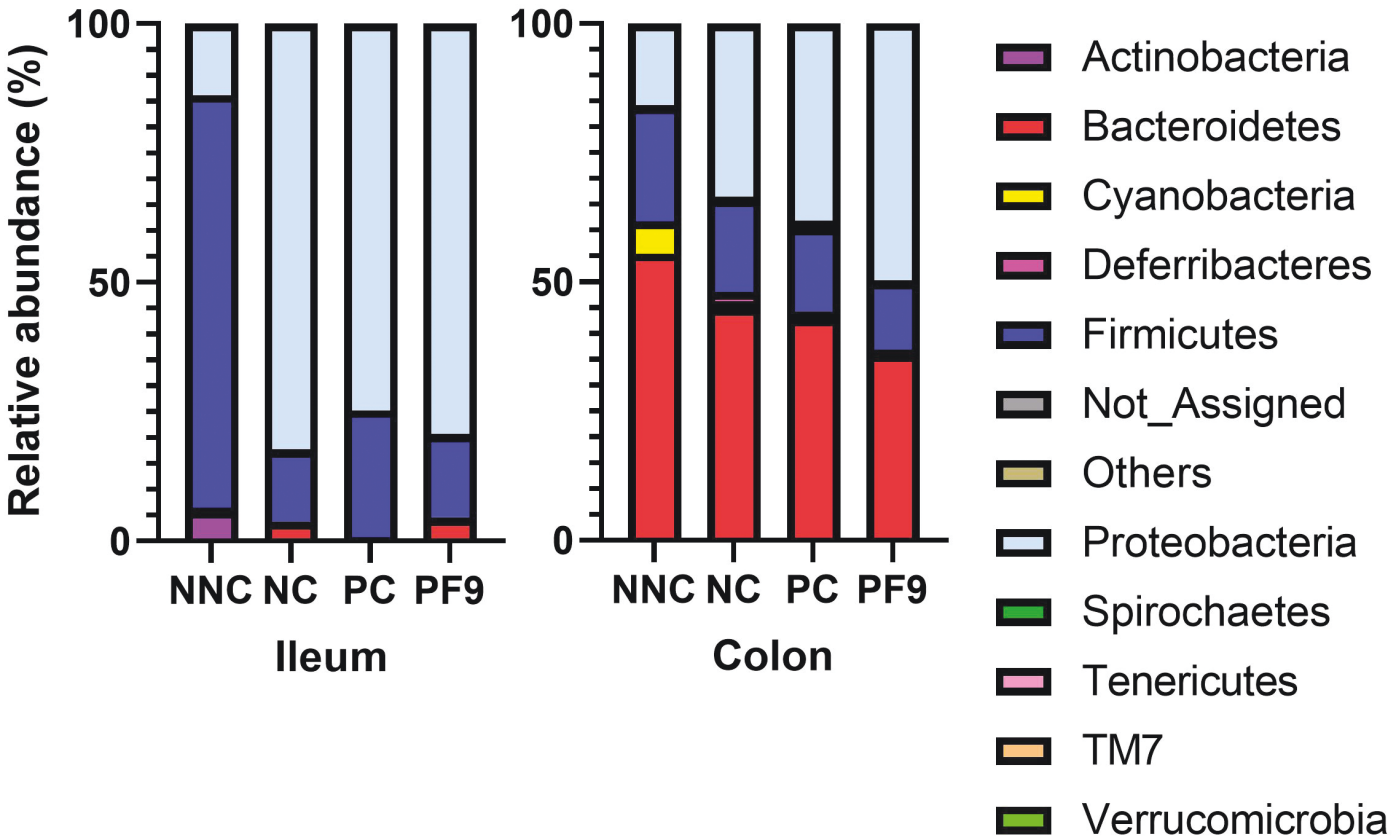
Stacked bar plot showing the relative abundance of bacterial phyla in the ileum and colon. Non-challenged negative control (NNC) group with basal diet and piglets gavaged with PBS; negative control (NC) group with basal diet and piglets challenged with enterotoxigenic *E. coli* (ETEC) F4; positive control (PC) group with basal diet + 80 mg·kg^−1^ of avilamycin and piglets challenged with ETEC F4; PF9 group with basal diet + 2.5 × 10^9^ CFU·kg^−1^ diet of *B. licheniformis* and piglets challenged with ETEC F4.

**Figure 6.**
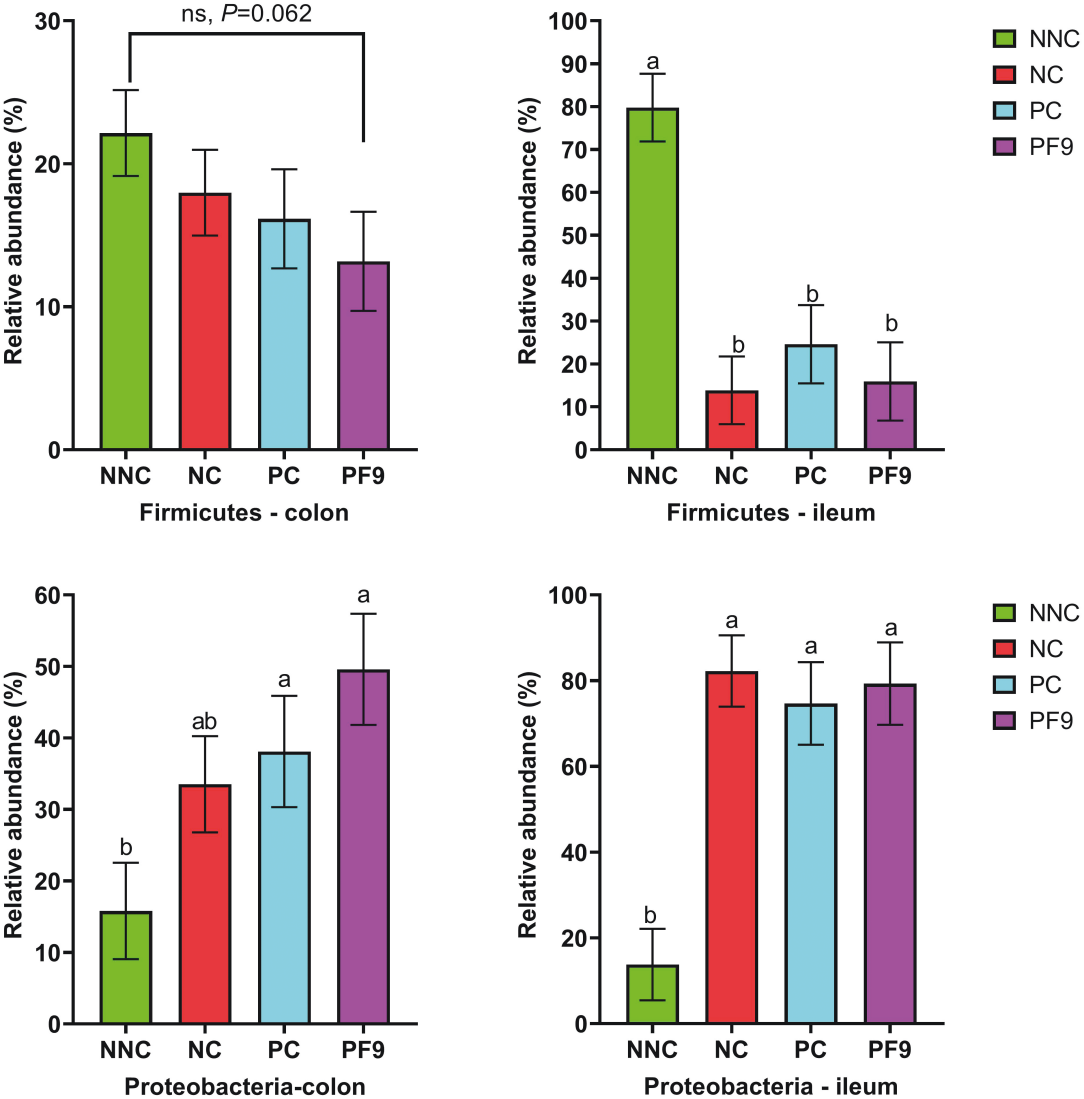
Bar plot showing the relative abundance of bacterial phylum in ileum and colon. ^a,b^Means without a common superscript are different (*P* < 0.05). Non-challenged negative control (NNC) group with basal diet and piglets gavaged with PBS; negative control (NC) group with basal diet and piglets challenged with enterotoxigenic *E. coli* (ETEC) F4; positive control (PC) group with basal diet + 80 mg·kg^−1^ of avilamycin and piglets challenged with ETEC F4; PF9 group with basal diet + 2.5 × 10^9^ CFU·kg^−1^ diet of *B. licheniformis* and piglets challenged with ETEC F4.

**Figure 7.**
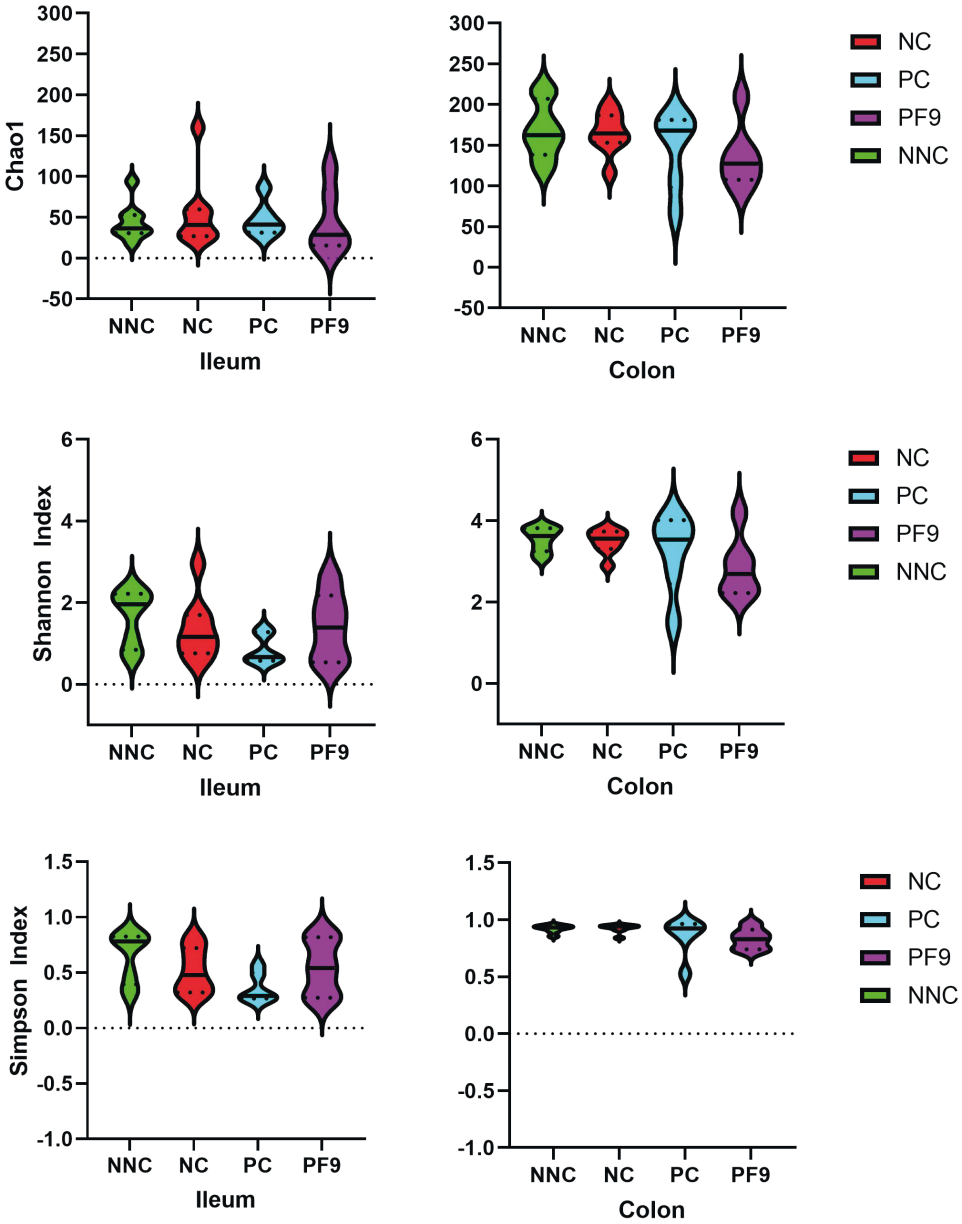
Alpha diversity as indicated by Chao1, Shannon, and Simpson index in digesta of ileum and colon. Non-challenged negative control (NNC) group with basal diet and piglets gavaged with PBS; negative control (NC) group with basal diet and piglets challenged with enterotoxigenic *E. coli* (ETEC) F4; positive control (PC) group with basal diet + 80 mg·kg^−1^ of avilamycin and piglets challenged with ETEC F4; PF9 group with basal diet + 2.5 × 10^9^ CFU·kg^−1^ diet of *B. licheniformis* and piglets challenged with ETEC F4.

**Figure 8.**
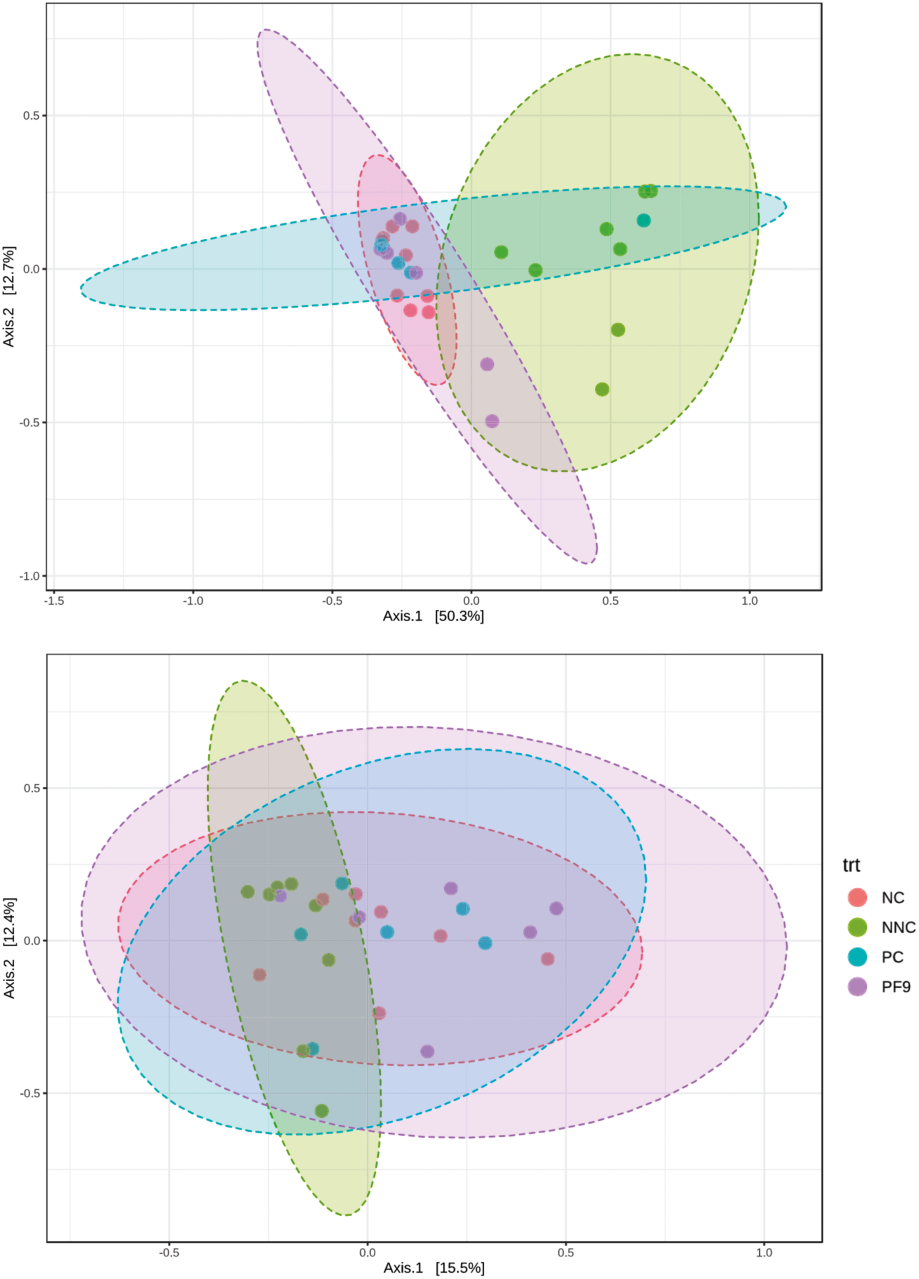
PCA plot of beta diversity of microbiota in digesta of ileum and colon based on Bray–Curtis dissimilarities. Non-challenged negative control (NNC) group with basal diet and piglets gavaged with PBS; negative control (NC) group with basal diet and piglets challenged with enterotoxigenic *E. coli* (ETEC) F4; positive control (PC) group with basal diet + 80 mg·kg^−1^ of avilamycin and piglets challenged with ETEC F4; PF9 group with basal diet + 2.5 × 10^9^ CFU·kg^−1^ diet of *B. licheniformis* and piglets challenged with ETEC F4.

In the current study, 3,906,109 qualified reads in total were obtained. A mean of 69,751 reads per sample and 2,862 operational taxonomic units in total were identified. The top phyla in the ileal digesta of non-challenged pigs were Firmicutes, which was replaced by Proteobacteria in challenged pigs. The colonic digesta was dominated by Bacteroidetes, Firmicutes, and Proteobacteria (Fig. 5). Piglets supplemented with *B. licheniformis* PF9 had a lower relative abundance of Bacteroidetes in the colon than piglets in the NNC group (*P* < 0.05). Non-challenged piglets had a higher relative abundance of Firmicutes in the ileum than the challenged piglets (*P* < 0.05); however, a lower relative abundance of Proteobacteria (*P* < 0.05) in the ileum and colon was observed in the NC group only (Fig. 6). For alpha diversity (Chao1, Shannon, and Simpson indices), there was no significant difference (*P* > 0.05) in the ileum and colon among all treatments (Fig. 7). No significant difference (*P* > 0.05) was found in the beta diversity (Bray–Curtis distance) of ileal and colon digesta among treatments (Fig. 8).

## DISCUSSION

The aim of this study was to characterize *B. licheniformis* PF9 and investigate its potential to relieve the negative effects of ETEC infection on weaned piglets, which impaired gut health including diarrhea and inflammation, by conducting both in vitro and in vivo studies.

The *B. licheniformis* PF9 showed tolerance to low pH and high bile salt in vitro, which provided the basic physiological foundation for the following in vivo animal trial. The same justification was also offered by the lifespan assay of *C. elegans*, in which *B. licheniformis* PF9 partially prevented the nematode from death caused by ETEC infection. Our previous studies have demonstrated that *Lactobacillus* was able to protect *C. elegans* against ETEC infection by suppressing the gene expression of STa and STb enterotoxins in ETEC (Zhou et al., 2014). In addition, the same *Lactobacillus* isolate could regulate the cell signaling of *C. elegans* to increase the production of defense molecules to combat ETEC infection (Zhou et al., 2018). It is unknown at present whether *B. licheniformis* PF9 has the same molecular mechanisms as the *Lactobacillus* isolate in the protection of *C. elegans* or not. Further studies are required to reach a conclusion.

The ETEC F4 challenge model in weaned piglets was established for inducing enteric infection (Opapeju et al., 2015). The ETEC F4 virulence and F4 fimbriae receptors in piglets are the two main factors leading to the pathogenesis of ETEC F4 (Luppi et al., 2016). Single nucleotide polymorphisms on F4 receptors MUC4 have been frequently used as the genetic markers for ETEC F4 susceptibility or resistance in pigs; therefore, the ETEC F4 susceptible piglets were chosen in the current study after gene screening of the susceptible alleles of MUC4 (Jørgensen et al., 2003).

In the present study, the supplementation of *B. licheniformis* PF9 did not affect the growth performance of piglets during the prechallenge period. The inoculation of ETEC F4 significantly decreased the ADG and gain:feed, which was similar to the findings of Ren et al. (2014). The potential mechanisms related to decreased ADG and gain:feed of piglets challenged with ETEC F4 have been attributed to reduced nutrient utilization efficiency (Choi et al., 2020); induced diarrhea (Sayan et al., 2018) and inflammatory responses (Xu et al., 2020); and reduced nutrients available for host growth because of ETEC F4 competition for available nutrients with the piglets (Mingmongkolchai and Panbangred, 2018). Inconsistent effects of the supplementation of *Bacillus* on pig growth performance have been reported. Pan et al. (2017) reported that the supplementation of probiotics containing *B. licheniformis* and *Saccharomyces cerevisiae* had positive effects on ADG and gain:feed in weaned piglets challenged with ETEC K88. However, Luise et al. (2019a) reported that *B. amyloliquefaciens* and *B. subtilis* did not show improvement in growth performance. These inconsistent results may be due to the differences in tested *Bacillus* isolates, their physiological status, ETEC strains (e.g., F4 and F18), and tested doses (de Lange et al., 2010) or experimental designs (e.g., the experimental period including pretreatment with the probiotic bacterium).

The higher diarrhea index score due to the ETEC challenge was an indicator showing the success of ETEC F4 infection in piglets (Luise et al., 2019b). In the present study, the supplementation of *B. licheniformis* PF9 reduced the severity of diarrhea to score values similar to those obtained with the antibiotic diet. It showed that both *B. licheniformis* PF9 and avilamycin mitigated the early stages of diarrhea caused by the ETEC F4 infection. Thus, *B. licheniformis* PF9 could be a promising alternative to antibiotics. This observation is consistent with a previous study described by Chen et al. (2021), which may in part be explained by enhanced gut barrier function with increased mRNA expression of the tight junction proteins ZO-1 and OCLN in the jejunum detected in the present study. This speculation was supported by the observation that the same isolate (*B. licheniformis* PF9) increased the expression levels of ZO-1 and OCLN in the ETEC F4-infected IPEC-J2 cells (Li et al., 2022). It has been well reported that the supplementation of probiotics can improve the gut barrier function of piglets (Wang et al., 2018; Yi et al., 2018).

To study the gut permeability of weaned piglets, 2 mg·mL^−1^ FITC-D70 in PBS was orally delivered to the piglets by gavage. In brief, FITC-D70 cannot pass through the epithelial barrier in the gut and be digested by gut digestive enzymes during normal healthy conditions; however, once the tight junction proteins have been affected by a pathogen, toxin, or inflammation, the FITC-D70 molecule could enter the blood circulation and could be detected in serum samples (Choi et al., 2020). The impairment of gut barrier function by the ETEC F4 challenge could increase the gut permeability with increased FITC-D70 flux, which affects several alternations in the morphology of the gut. It has been reported that the supplementation of *Bacillus subtilis* reduced paracellular permeability determined by measuring the flux of FITC-D4 across the jejunal mucosa (Kim et al., 2019). The inconsistent results in our current study may be due to the different strains.

The higher VH could indicate better nutrient digestion and absorption as the enterocytes located in the villus of piglets were critical in nutrient utilization (Chen et al., 2018). In the present study, the inoculation of ETEC F4 decreased the VH of the small intestine, which is consistent with a report by Choi et al. (2020). ETEC F4 adhering to the enterocyte brush border membrane of gut mucosa causing villous atrophy may be the possible reason for decreasing VH (Luppi, 2017). In the current study, the supplementation of *B. licheniformis* demonstrated a tendency to increase VH in the ileum when compared with the NC group, which is similar to a study from Zong et al. (2019). This may partially explain the increased relative mRNA abundance of the brush border digestive enzymes, APN and MGA, in *B. licheniformis* PF9-treated piglets.

In this study, the ETEC F4 infection significantly decreased the relative mRNA level of SGLT1 in the mid-jejunum, which were similar results to those found in a study by Choi et al. (2020). The SGLT1 plays an important role in the gut glucose transport system in pigs (Yang et al., 2011). The decreased SGLT1 may be associated with secreted toxins and villous atrophy from ETEC F4 (Wu et al., 2015). In the present study, the supplementation of *B. licheniformis* PF9 increased the relative mRNA expression of SGLT1 and EAAC1. These data could be supported by Cao et al. (2020) reporting that the increased expression of nutrient transporters in the intestine can reflect the strong absorption function of epithelial cells in the small intestine due to the supplementation of probiotics.

The diversity and composition of gut microbiota in piglets are strongly related to animal health conditions and nutrient compositions that are provided by animal diets (Guevarra et al., 2018). In the present study, the supplementation of *B. licheniformis* PF9 showed no impact on the microbiota diversity in the digesta of ileum and colon, which were similar results to those found in a study by He et al. (2020). Nonetheless, with findings that were inconsistent with those previously published by Duarte et al. (2020), the ETEC infection significantly increased the relative abundance of Proteobacteria and reduced the relative abundance of Firmicutes. This may be partially explained by the production of enterotoxins due to ETEC infection and secretion of fluid to the gut lumen (Wang et al., 2019), which creates an ideal environment for the growth of Proteobacteria (Gresse et al., 2017). The results from the current study indicated that the supplementation of *B. licheniformis* PF9 decreased the relative abundance of Bacteroidetes, which is consistent with the study reported by Cui et al. (2013).

In conclusion, results from the current study demonstrated that the heat-resistant *B. licheniformis* PF9 was tolerant to low pH and high bile salt. It provided protection to *C. elegans* against ETEC infection. The infection of ETEC F4 impaired growth performance, damaged gut morphology, induced diarrhea, and changed the gut bacterial population in weaned piglets. The supplementation of *B. licheniformis* PF9 reduced diarrhea score, increased the relative mRNA level of several nutrient transporters, the gut barrier-associated proteins, and digestive enzymes, and lowered the relative abundance of Bacteroidetes in the ETEC F4-challenged weaned piglets. These results suggest that *B. licheniformis* PF9 has the potential for improving pig gut health. Further studies are needed to evaluate the different dose of this isolate and a cocktail of probiotics containing different probiotic candidates for their potential in controlling enteric infections and improving the gut health and performance of pigs.

## References

[CIT0001] Bokulich, N. A., B. D. Kaehler, J. R. Rideout, M. Dillon, E. Bolyen, R. Knight, G. A. Huttley, and J. G. Caporaso. 2018. Optimizing taxonomic classification of marker-gene amplicon sequences with QIIME 2’s q2-feature-classifier plugin. Microbiome. 6:1–17. doi:10.1186/s40168-018-0470-z 29773078 PMC5956843

[CIT0002] Bolyen, E., J. R. Rideout, M. R. Dillon, N. A. Bokulich, C. C. Abnet, G. A. Al-Ghalith, H. Alexander, E. J. Alm, M. Arumugam, F. Asnicar, et al. 2019. Reproducible, interactive, scalable and extensible microbiome data science using QIIME 2. Nat. Biotechnol. 37:852–857. doi:10.1038/s41587-019-0209-9 31341288 PMC7015180

[CIT0003] Breger, J., B. B. Fuchs, G. Aperis, T. I. Moy, F. M. Ausubel, and E. Mylonakis. 2007. Antifungal chemical compounds identified using a *C. elegans* pathogenicity assay. PLoS Pathog. 3:e18. doi:10.1371/journal.ppat.0030018 17274686 PMC1790726

[CIT0004] Callahan, B. J., P. J. McMurdie, M. J. Rosen, A. W. Han, A. J. A. Johnson, and S. P. Holmes. 2016. DADA2: high-resolution sample inference from Illumina amplicon data. Nat. Methods. 13:581–583. doi:10.1038/nmeth.3869 27214047 PMC4927377

[CIT0005] Canadian Council on Animal Care (CCAC). 2009. CCAC guideline on: the care and use of farm animals in research, teaching and testing. CCAC, Ottawa, ON.

[CIT0006] Cao, X., L. Tang, Z. Zeng, B. Wang, Y. Zhou, Q. Wang, P. Zou, and W. Li. 2020. Effects of probiotics BaSC06 on intestinal digestion and absorption, antioxidant capacity, microbiota composition, and macrophage polarization in pigs for fattening. Front. Vet. Sci. 7:570593. doi:10.3389/fvets.2020.570593 33240950 PMC7677304

[CIT0007] Caporaso, J. G., J. Kuczynski, J. Stombaugh, K. Bittinger, F. D. Bushman, E. K. Costello, N. Fierer, A. G. Peña, J. K. Goodrich, J. I. Gordon, et al. 2010. correspondence QIIME allows analysis of high- throughput community sequencing data Intensity normalization improves color calling in SOLiD sequencing. Nat. Publ. Gr. 7:335–336. doi:10.1038/nmeth.f.303 PMC3156573 20383131

[CIT0008] Caporaso, J. G., C. L. Lauber, W. A. Walters, D. Berg-Lyons, J. Huntley, N. Fierer, S. M. Owens, J. Betley, L. Fraser, M. Bauer, et al. 2012. Ultra-high-throughput microbial community analysis on the Illumina HiSeq and MiSeq platforms. ISME J. 6:1621–1624. doi:10.1038/ismej.2012.8 22402401 PMC3400413

[CIT0009] Chen, L., S. Li, J. Zheng, W. Li, X. Jiang, X. Zhao, J. Li, L. Che, Y. Lin, S. Xu, et al. 2018. Effects of dietary *Clostridium butyricum* supplementation on growth performance, intestinal development, and immune response of weaned piglets challenged with lipopolysaccharide. J. Anim. Sci. Biotechnol. 9:1–14. doi:10.1186/s40104-018-0275-8 30159141 PMC6106813

[CIT0010] Chen, Y., S. Liu, C. Yu, P. Azevedo, S. Liu, O. K, J. Gong, Y. Hou, and C. Yang. 2021. Evaluating the effectiveness of *Lactobacillus zeae* against enterotoxigenic *Escherichia coli* F4 infection in an in vitro porcine intestinal epithelial cell model. ACS Food Sci. Technol. 1:215–228. doi:10.1021/acsfoodscitech.0c00069

[CIT0011] Choi, J., L. Wang, S. Liu, P. Lu, X. Zhao, H. Liu, L. Lahaye, E. Santin, S. Liu, M. Nyachoti, et al. 2020. Effects of a microencapsulated formula of organic acids and essential oils on nutrient absorption, immunity, gut barrier function, and abundance of enterotoxigenic *Escherichia coli* F4 in weaned piglets challenged with *E. coli* F4. J. Anim. Sci. 98:skaa259. doi:10.1093/jas/skaa259 32780110 PMC7526869

[CIT0012] Cui, C., C. J. Shen, G. Jia, and K. N. Wang. 2013. Effect of dietary *Bacillus subtilis* on proportion of Bacteroidetes and Firmicutes in swine intestine and lipid metabolism. Genet. Mol. Res. 12:1766–1776. doi:10.4238/2013.May.23.1 23765983

[CIT0013] Dahlqvist, A. 1964. Method for assay of intestinal disaccharidases. Anal. Biochem. 7:18–25. doi:10.1016/0003-2697(64)90115-0 14106916

[CIT0026] de Lange, C. F. M., J. Pluske, J. Gong, and C. M. Nyachoti. 2010. Strategic use of feed ingredients and feed additives to stimulate gut health and development in young pigs. Livest. Sci. 134:124–134. doi:10.1016/j.livsci.2010.06.117

[CIT0014] Duarte, M. E., J. Tyus, and S. W. Kim. 2020. Synbiotic effects of enzyme and probiotics on intestinal health and growth of newly weaned pigs challenged with enterotoxigenic F18+ *Escherichia coli*. Front. Vet. Sci. 7:1–13. doi:10.3389/fvets.2020.00573 33033721 PMC7509054

[CIT0015] Ekakoro, J. E., M. Caldwell, E. B. Strand, and C. C. Okafor. 2019. Perceptions of Tennessee cattle producers regarding the veterinary feed directive. PLoS One. 14:e0217773. doi:10.1371/journal.pone.0217773 31150500 PMC6544306

[CIT0016] Gresse, R., F. Chaucheyras-Durand, M. A. Fleury, T. Van de Wiele, E. Forano, and S. Blanquet-Diot. 2017. Gut microbiota dysbiosis in postweaning piglets: understanding the keys to health. Trends Microbiol. 25:851–873. doi:10.1016/j.tim.2017.05.004 28602521

[CIT0017] Guevarra, R. B., S. H. Hong, J. H. Cho, B. R. Kim, J. Shin, J. H. Lee, B. N. Kang, Y. H. Kim, S. Wattanaphansak, R. E. Isaacson, et al. 2018. The dynamics of the piglet gut microbiome during the weaning transition in association with health and nutrition. J. Anim. Sci. Biotechnol. 9:1–9. doi:10.1186/s40104-018-0269-6 30069307 PMC6065057

[CIT0018] He, Y., C. Jinno, K. Kim, Z. Wu, B. Tan, X. Li, R. Whelan, and Y. Liu. 2020. Dietary *Bacillus* spp. enhanced growth and disease resistance of weaned pigs by modulating intestinal microbiota and systemic immunity. J. Anim. Sci. Biotechnol. 11:1–19. doi:10.1186/s40104-020-00498-3 32944236 PMC7491085

[CIT0019] Horng, Y. B., Y. H. Yu, A. Dybus, F. S. H. Hsiao, and Y. H. Cheng. 2019. Antibacterial activity of *Bacillus* species-derived surfactin on *Brachyspira hyodysenteriae* and *Clostridium perfringens*. AMB Express. 9:1–9. doi:10.1186/s13568-019-0914-2 31754906 PMC6872690

[CIT0020] Hübscher, G., and G. R. West. 1965. Specific assays of some phosphatases in subcellular fractions of small intestinal mucosa. Nature. 205:799–800. doi:10.1038/205799a0 14292314

[CIT0021] Jensen, G. M., K. Frydendahl, O. Svendsen, C. B. Jørgensen, S. Cirera, M. Fredholm, J. P. Nielsen, and K. Møller. 2006. Experimental infection with *Escherichia coli* O149:F4ac in weaned piglets. Vet. Microbiol. 115:243–249. doi:10.1016/j.vetmic.2006.01.002 16466864

[CIT0022] Jørgensen, C. B., S. Cirera, S. I. Anderson, A. L. Archibald, T. Raudsepp, B. Chowdhary, I. Edfors-Lilja, L. Andersson, and M. Fredholm. 2003. Linkage and comparative mapping of the locus controlling susceptibility towards *E. coli* F4ab/ac diarrhoea in pigs. Cytogenet. Genome Res. 102:157–162. doi:10.1159/000075742 14970696

[CIT0023] Kim, K., Y. He, X. Xiong, A. Ehrlich, X. Li, H. Raybould, E. R. Atwill, E. A. Maga, J. Jørgensen, and Y. Liu. 2019. Dietary supplementation of *Bacillus subtilis* influenced intestinal health of weaned pigs experimentally infected with a pathogenic *E. coli*. J. Anim. Sci. Biotechnol. 10:1–12. doi:10.1186/s40104-019-0364-330651985

[CIT0024] Koo, B., D. Bustamante-García, J. W. Kim, and C. M. Nyachoti. 2020. Health-promoting effects of Lactobacillus-fermented barley in weaned pigs challenged with *Escherichia coli* K88 +. Animal. 14:39–49. doi:10.1017/S1751731119001939 31426877

[CIT0025] Lackeyram, D., Y. Mine, T. Widowski, T. Archbold, and M. Z. Fan. 2012. The in vivo infusion of hydrogen peroxide induces oxidative stress and differentially affects the activities of small intestinal carbohydrate digestive enzymes in the neonatal pig. J. Anim. Sci. 90:418–420. doi:10.2527/jas.54011 23365398

[CIT0027] Lardé, H., D. Francoz, J. P. Roy, J. Massé, M. Archambault, M. Paradis, and S. Dufour. 2021. Comparison of quantification methods to estimate farm-level usage of antimicrobials other than in medicated feed in dairy farms from Québec, Canada. Microorganisms. 9:1106. doi:10.3390/microorganisms9051106 34576729 PMC8471653

[CIT0028] Lee, S. I., and K. S. Kang. 2017. Function of capric acid in cyclophosphamide-induced intestinal inflammation, oxidative stress, and barrier function in pigs. Sci. Rep. 7:1–12. doi:10.1038/s41598-017-16561-5 29184078 PMC5705592

[CIT0029] Li, Q., L. Li, Y. Chen, C. Yu, P. Azevedo, J. Gong, and C. Yang. 2022. *Bacillus licheniformis* PF9 improves barrier function and alleviates inammatory responses against enterotoxigenic *Escherichia coli* F4 infection in the porcine intestinal epithelial cells. J. Animal Sci. Biotechnol. 13:86. doi:10.1186/s40104-022-00746-8 PMC9264548 35799262

[CIT0030] Liao, S. F., and M. Nyachoti. 2017. Using probiotics to improve swine gut health and nutrient utilization. Anim. Nutr. 3:331–343. doi:10.1016/j.aninu.2017.06.007 29767089 PMC5941265

[CIT0031] Liu, Y., C. D. Espinosa, J. J. Abelilla, G. A. Casas, L. V. Lagos, S. A. Lee, W. B. Kwon, J. K. Mathai, D. M. D. L. Navarro, N. W. Jaworski, et al. 2018. Non-antibiotic feed additives in diets for pigs: a review. Anim. Nutr. 4:113–125. doi:10.1016/j.aninu.2018.01.007 30140751 PMC6103469

[CIT0032] Livak, K. J., and T. D. Schmittgen. 2001. Analysis of relative gene expression data using real-time quantitative PCR and the 2-ΔΔCT method. Methods 25:402–408. doi:10.1006/meth.2001.1262 11846609

[CIT0033] Luise, D., M. Bertocchi, V. Motta, C. Salvarani, P. Bosi, A. Luppi, F. Fanelli, M. Mazzoni, I. Archetti, G. Maiorano, et al. 2019a. *Bacillus* sp. probiotic supplementation diminish the *Escherichia coli* F4ac infection in susceptible weaned pigs by influencing the intestinal immune response, intestinal microbiota and blood metabolomics. J. Anim. Sci. Biotechnol. 10:1–16. doi:10.1186/s40104-019-0380-3 31528339 PMC6740008

[CIT0034] Luise, D., C. Lauridsen, P. Bosi, and P. Trevisi. 2019b. Methodology and application of *Escherichia coli* F4 and F18 encoding infection models in post-weaning pigs. J. Anim. Sci. Biotechnol. 10:1–20. doi:10.1186/s40104-019-0352-7 31210932 PMC6567477

[CIT0035] Luppi, A. 2017. Swine enteric colibacillosis: diagnosis, therapy and antimicrobial resistance. Porc. Heal. Manag. 3:1–18. doi:10.1186/s40813-017-0063-4 PMC5547460 28794894

[CIT0036] Luppi, A., M. Gibellini, T. Gin, F. Vangroenweghe, V. Vandenbroucke, R. Bauerfeind, P. Bonilauri, G. Labarque, and A. Hidalgo. 2016. Prevalence of virulence factors in enterotoxigenic *Escherichia coli* isolated from pigs with post-weaning diarrhoea in Europe. Porc. Heal. Manag. 2:1–6. doi:10.1186/s40813-016-0039-9 PMC5382528 28405446

[CIT0037] Maroux, S., D. Louvard, and J. Barath. 1973. The aminopeptidase from hog intestinal brush border. Biochim. Biophys. Acta. 321:282–295. doi:10.1016/0005-2744(73)90083-1. 4750768

[CIT0038] Marquardt, R. R., L. Z. Jin, J. W. Kim, L. Fang, A. A. Frohlich, and S. K. Baidoo. 1999. Passive protective effect of egg-yolk antibodies against enterotoxigenic *Escherichia coli* K88+ infection in neonatal and early-weaned piglets. FEMS Immunol. Med. Microbiol. 23:283–288. doi:10.1111/j.1574-695X.1999.tb01249.x 10225287

[CIT0039] Martin, M. J., S. E. Thottathil, and T. B. Newman. 2015. Antibiotics overuse in animal agriculture: a call to action for health care providers. Am. J. Public Health. 105:2409–2410. doi:10.2105/AJPH.2015.302870 26469675 PMC4638249

[CIT0040] Matsumoto, H., M. Miyagawa, S. Takahashi, R. Shima, and T. Oosumi. 2020. Improvement of the enterotoxigenic *Escherichia coli* infection model for post-weaning diarrhea by controlling for bacterial adhesion, pig breed and MUC4 genotype. Vet. Sci. 7:106. doi:10.3390/vetsci7030106 32784676 PMC7557722

[CIT0041] Mingmongkolchai, S., and W. Panbangred. 2018. *Bacillus* probiotics: an alternative to antibiotics for livestock production. J. Appl. Microbiol. 124:1334–1346. doi:10.1111/jam.13690 29316021

[CIT0042] Noamani, B. N., J. M. Fairbrother, and C. L. Gyles. 2003. Virulence genes of O149 enterotoxigenic *Escherichia coli* from outbreaks of postweaning diarrhea in pigs. Vet. Microbiol. 97:87–101. doi:10.1016/j.vetmic.2003.08.006 14637041

[CIT0070] NRC. 2012. *Nutrient requirements of swine*. Washington (DC): The National Academies Press.

[CIT0043] Olajide, A. M., S. Chen, and G. LaPointe. 2021. Markers to rapidly distinguish *Bacillus paralicheniformis* from the very close relative, *Bacillus licheniformis*. Front. Microbiol. 11:596828. doi:10.3389/fmicb.2020.596828 33505369 PMC7829221

[CIT0044] Omonijo, F. A., S. Kim, T. Guo, Q. Wang, J. Gong, L. Lahaye, J. C. Bodin, M. Nyachoti, S. Liu, and C. Yang. 2018. Development of novel microparticles for effective delivery of thymol and lauric acid to pig intestinal tract. J. Agric. Food Chem. 66:9608–9615. doi:10.1021/acs.jafc.8b02808 30141924

[CIT0045] Omonijo, F. A., S. Liu, Q. Hui, H. Zhang, L. Lahaye, J. C. Bodin, J. Gong, M. Nyachoti, and C. Yang. 2019. Thymol improves barrier function and attenuates inflammatory responses in porcine intestinal epithelial cells during lipopolysaccharide (LPS)-induced inflammation. J. Agric. Food Chem. 67:615–624. doi:10.1021/acs.jafc.8b05480 30567427

[CIT0046] Opapeju, F. O., J. C. Rodriguez-Lecompte, M. Rademacher, D. O. Krause, and C. M. Nyachoti. 2015. Low crude protein diets modulate intestinal responses in weaned pigs challenged with *Escherichia coli* K88. Can. J. Anim. Sci. 95:71–78. doi:10.4141/cjas-2014-071

[CIT0047] Pan, L., P. F. Zhao, X. K. Ma, Q. H. Shang, Y. T. Xu, S. F. Long, Y. Wu, F. M. Yuan, and X. S. Piao. 2017. Probiotic supplementation protects weaned pigs against enterotoxigenic *Escherichia coli* k88 challenge and improves performance similar to antibiotics. J. Anim. Sci. 95:2627–2639. doi:10.2527/jas.2016.1243 28727032

[CIT0048] Quast, C., E. Pruesse, P. Yilmaz, J. Gerken, T. Schweer, P. Yarza, J. Peplies, and F. O. Glöckner. 2013. The SILVA ribosomal RNA gene database project: Improved data processing and web-based tools. Nucleic Acids Res. 41:D590–D596. doi:10.1093/nar/gks1219 23193283 PMC3531112

[CIT0049] Ren, M., X. T. Liu, X. Wang, G. J. Zhang, S. Y. Qiao, and X. F. Zeng. 2014. Increased levels of standardized ileal digestible threonine attenuate intestinal damage and immune responses in *Escherichia coli* K88+ challenged weaned piglets. Anim. Feed Sci. Technol. 195:67–75. doi:10.1016/j.anifeedsci.2014.05.013

[CIT0050] Rhouma, M., J. M. Fairbrother, F. Beaudry, and A. Letellier. 2017. Post weaning diarrhea in pigs: risk factors and non-colistin-based control strategies. Acta Vet. Scand. 59:1–19. doi:10.1186/s13028-017-0299-7 28526080 PMC5437690

[CIT0051] Rozs, M., L. Manczinger, C. Vágvölgyi, and F. Kevei. 2001. Secretion of a trypsin-like thiol protease by a new keratinolytic strain of *Bacillus licheniformis*. FEMS Microbiol. Lett. 205:221–224. doi:10.1111/j.1574-6968.2001.tb10951.x 11750806

[CIT0052] Sayan, H., P. Assavacheep, K. Angkanaporn, and A. Assavacheep. 2018. Effect of *Lactobacillus salivarius* on growth performance, diarrhea incidence, fecal bacterial population and intestinal morphology of suckling pigs challenged with F4+ enterotoxigenic *Escherichia coli*. Asian-Australas. J. Anim. Sci. 31:1308–1314. doi:10.5713/ajas.17.0746 29642683 PMC6043459

[CIT0053] Thoms, U. 2012. Between promise and threat: antibiotics in foods in West Germany 1950-1980. NTM. 20:181–214. doi:10.1007/s00048-012-0073-x 22941180

[CIT0054] Tohno, M., T. Shimosato, H. Kitazawa, S. Katoh, I. D. Iliev, T. Kimura, Y. Kawai, K. Watanabe, H. Aso, T. Yamaguchi, et al. 2005. Toll-like receptor 2 is expressed on the intestinal M cells in swine. Biochem. Biophys. Res. Commun. 330:547–554. doi:10.1016/j.bbrc.2005.03.017 15796917

[CIT0055] Truett, G. E., P. Heeger, R. L. Mynatt, A. A. Truett, J. A. Walker, and M. L. Warman. 2000. Preparation of PCR-quality mouse genomic DNA with hot sodium hydroxide and tris (HotSHOT). Biotechniques. 29:52, 54–52, 54. doi:10.2144/00291bm09 10907076

[CIT0056] Wang, H., Z. Zhong, Y. Luo, E. Cox, and B. Devriendt. 2019. Heat-stable enterotoxins of enterotoxigenic *Escherichia coli* and their impact on host immunity. Toxins (Basel). 11:24–12. doi:10.3390/toxins11010024 30626031 PMC6356903

[CIT0057] Wang, J., H. Ji, S. Wang, H. Liu, W. Zhang, D. Zhang, and Y. Wang. 2018. Probiotic *Lactobacillus plantarum* promotes intestinal barrier function by strengthening the epithelium and modulating gut microbiota. Front. Microbiol. 9:1–14. doi:10.3389/fmicb.2018.01953 30197632 PMC6117384

[CIT0058] Wang, Q., J. Gong, X. Huang, H. Yu, and F. Xue. 2009. In vitro evaluation of the activity of microencapsulated carvacrol against *Escherichia coli* with K88 pili. J. Appl. Microbiol. 107:1781–1788. doi:10.1111/j.1365-2672.2009.04374.x 19645769

[CIT0059] Weinman, S. A., M. W. Carruth, and P. A. Dawson. 1998. Bile acid uptake via the human apical sodium-bile acid cotransporter is electrogenic. J. Biol. Chem. 273:34691–34695. doi:10.1074/jbc.273.52.34691 9856990

[CIT0060] Wu, L., P. Liao, L. He, W. Ren, J. Yin, J. Duan, and T. Li. 2015. Growth performance, serum biochemical profile, jejunal morphology, and the expression of nutrients transporter genes in deoxynivalenol (DON)-challenged growing pigs. BMC Vet. Res. 11:1–10. doi:10.1186/s12917-015-0449-y 26138080 PMC4490653

[CIT0061] Xu, Y., L. Lahaye, Z. He, J. Zhang, C. Yang, and X. Piao. 2020. Micro-encapsulated essential oils and organic acids combination improves intestinal barrier function, inflammatory responses and microbiota of weaned piglets challenged with enterotoxigenic *Escherichia coli* F4 (K88+). Anim. Nutr. 6:269–277. doi:10.1016/j.aninu.2020.04.004 33005760 PMC7503083

[CIT0062] Yang, C. 2011. Expression of porcine intestinal nutrient transporters along crypt-villus axis and during postnatal development. Guelph, Canada: University of Guleph. 359.

[CIT0063] Yang, C., D. M. Albin, Z. Wang, B. Stoll, D. Lackeyram, K. C. Swanson, Y. Yin, K. A. Tappenden, Y. Mine, R. Y. Yada, et al. 2011. Apical Na+-d-glucose cotransporter 1 (SGLT1) activity and protein abundance are expressed along the jejunal crypt-villus axis in the neonatal pig. Am. J. Physiol. Gastrointest. Liver Physiol. 300:G60–G70. doi:10.1152/ajpgi.00208.2010 21030609 PMC3025512

[CIT0064] Yang, C., X. Yang, D. Lackeyram, T. C. Rideout, Z. Wang, B. Stoll, Y. Yin, D. G. Burrin, and M. Z. Fan. 2016. Expression of apical Na+–l-glutamine co-transport activity, B0-system neutral amino acid co-transporter (B0AT1) and angiotensin-converting enzyme 2 along the jejunal crypt–villus axis in young pigs fed a liquid formula. Amino Acids. 48:1491–1508. doi:10.1007/s00726-016-2210-7 26984322

[CIT0065] Yang, X., J. Brisbin, H. Yu, Q. Wang, F. Yin, Y. Zhang, P. Sabour, S. Sharif, and J. Gong. 2014. Selected lactic acid-producing bacterial isolates with the capacity to reduce salmonella translocation and virulence gene expression in chickens. PLoS One. 9:e93022. doi:10.1371/journal.pone.0093022 24728092 PMC3984083

[CIT0066] Yi, H., L. Wang, Y. Xiong, X. Wen, Z. Wang, X. Yang, K. Gao, and Z. Jiang. 2018. Effects of *Lactobacillus reuteri* LR1 on the growth performance, intestinal morphology, and intestinal barrier function in weaned pigs. J. Anim. Sci. 96:2342–2351. doi:10.1093/jas/sky129 29659876 PMC6095392

[CIT0067] Zhou, M., X. Liu, H. Yu, X. Yin, S. P. Nie, M. Y. Xie, W. Chen, and J. Gong. 2018. Cell signaling of *Caenorhabditis elegans* in response to enterotoxigenic *Escherichia coli* infection and *Lactobacillus zeae* protection. Front. Immunol. 9:1–11. doi:10.3389/fimmu.2018.01745 30250464 PMC6139356

[CIT0068] Zhou, M., H. Yu, X. Yin, P. M. Sabour, W. Chen, and J. Gong. 2014. *Lactobacillus zeae* protects *Caenorhabditis elegans* from enterotoxigenic *Escherichia coli*-caused death by inhibiting enterotoxin gene expression of the pathogen. PLoS One. 9:e89004–e89010. doi:10.1371/journal.pone.0089004 24558463 PMC3928337

[CIT0069] Zong, X., T. H. Wang, Z. Q. Lu, D. G. Song, J. Zhao, and Y. Z. Wang. 2019. Effects of *Clostridium butyricum* or in combination with *Bacillus licheniformis* on the growth performance, blood indexes, and intestinal barrier function of weanling piglets. Livest. Sci. 220:137–142. doi:10.1016/j.livsci.2018.12.024

